# Comparative analysis of continuum angiogenesis models

**DOI:** 10.1007/s00285-021-01570-w

**Published:** 2021-02-23

**Authors:** W. Duncan Martinson, Hirokazu Ninomiya, Helen M. Byrne, Philip K. Maini

**Affiliations:** 1 Wolfson Centre for Mathematical Biology, Mathematical Institute, University of Oxford, Woodstock Road, Oxford, OX2 6GG United Kingdom; 2 Meiji University, School of Interdisciplinary Mathematical Sciences, 4-21-1 Nakano, Nakano-ku, Tokyo, 164-8525 Japan

**Keywords:** Snail-trail model, Perturbation methods, Coarse-grained models, Discrete-to-continuum modelling, angiogenesis, Agent-based modelling, 92C17, 92C37, 35Q92, 41A60

## Abstract

Although discrete approaches are increasingly employed to model biological phenomena, it remains unclear how complex, population-level behaviours in such frameworks arise from the rules used to represent interactions between individuals. Discrete-to-continuum approaches, which are used to derive systems of coarse-grained equations describing the mean-field dynamics of a microscopic model, can provide insight into such emergent behaviour. Coarse-grained models often contain nonlinear terms that depend on the microscopic rules of the discrete framework, however, and such nonlinearities can make a model difficult to mathematically analyse. By contrast, models developed using phenomenological approaches are typically easier to investigate but have a more obscure connection to the underlying microscopic system. To our knowledge, there has been little work done to compare solutions of phenomenological and coarse-grained models. Here we address this problem in the context of angiogenesis (the creation of new blood vessels from existing vasculature). We compare asymptotic solutions of a classical, phenomenological “snail-trail” model for angiogenesis to solutions of a nonlinear system of partial differential equations (PDEs) derived via a systematic coarse-graining procedure (Pillay et al. in Phys Rev E 95(1):012410, 2017. https://doi.org/10.1103/PhysRevE.95.012410). For distinguished parameter regimes corresponding to chemotaxis-dominated cell movement and low branching rates, both continuum models reduce at leading order to identical PDEs within the domain interior. Numerical and analytical results confirm that pointwise differences between solutions to the two continuum models are small if these conditions hold, and demonstrate how perturbation methods can be used to determine when a phenomenological model provides a good approximation to a more detailed coarse-grained system for the same biological process.

**Supplementary Information:**

The online version contains supplementary material available at 10.1007/s00285-021-01570-w.

## Introduction

Continuum and discrete approaches are widely used to develop theoretical models for biological systems. In the former framework, one approximates quantities of interest using continuous variables and measures their evolution in space and/or time via differential equations. By contrast, discrete approaches describe the behaviour of individual “agents” (such as cells or molecules) in simulations (An et al. 2009; Van Liedekerke et al. 2015; Metzcar et al. 2019). These two modelling frameworks are not mutually exclusive: for instance, “hybrid models” use continuous approaches to represent certain quantities of interest (e.g., the concentration of a drug), but apply discrete methods for other quantities such as cells (Rejniak and Anderson 2011). Hybrid and discrete models for a biological process can typically incorporate more faithful representations of underlying cellular and/or molecular mechanisms than phenomenological continuum systems, and they are becoming increasingly prevalent in the mathematical biology community (Van Liedekerke et al. 2015; Metzcar et al. 2019).

Despite the advantages and wide use of agent-based models (ABMs), we still have a poor understanding of how different parameter values and agent interaction rules affect their solutions over large spatial and temporal scales. Kursawe et al. (2017), for example, found that results from a particular discrete model (the vertex model) are sensitive to the manner in which it is numerically implemented. Another study by Osborne et al. (2017) compared discrete approaches for modelling cell proliferation, adhesion, and signalling using a single computational framework. The models that they investigated (which included cellular automata, the Cellular Potts model, the overlapping spheres model, Voronoi tessellations, and the vertex model) did not always generate equivalent summary statistics; their work thereby illustrated how inferred biological conclusions may depend on the modelling approach used to generate results. Although such studies represent a much needed first step for evaluating discrete approaches and their underlying rules, it is challenging to gain rigorous insight into the general behaviour of ABMs unless there is a common mathematical framework in which to analyse them.

Coarse-grained macroscopic partial differential equations (PDEs) represent a possible candidate for this framework. They are constructed from discrete-to-continuum derivations, rather than from phenomenological arguments, and describe how the average distribution of agents in a discrete model evolves over time and space (Othmer et al. 1988; Baker et al. 2010; Simpson and Baker 2011; Markham et al. 2013; Bonilla et al. 2016; Dyson and Baker 2015; Spill et al. 2015; Buttenschön et al. 2018; Motsch and Peurichard 2018; Chaplain et al. 2020). Since continuum models are often easier to analyse and simulate than discrete ones, they can be used to (indirectly) determine how the microscopic rules of a discrete model generate complex collective dynamics. However, mean-field models derived from discrete-to-continuum approaches tend to be highly nonlinear, and the degree of nonlinearity depends on the rules in the underlying discrete model (Simpson et al. 2009; Penington et al. 2011; Bruna and Chapman 2012; Penington et al. 2014; Pillay et al. 2018). To our knowledge, little recent attention has focussed on the systematic comparison of nonlinear mean-field models derived from discrete-to-continuum approaches (Horstmann et al. 2004). In this article, we aim to shed light on the connection between coarse-grained and phenomenological models by analysing solutions from two particular continuum systems for angiogenesis (the growth of new blood vessels from pre-existing vasculature). Angiogenesis is implicated in many essential biological processes, such as wound healing and tissue growth, because the resulting blood vessels provide a source of nutrients for damaged and/or developing tissue (Potente et al. 2011; Viallard and Larrivée 2017; Duran et al. 2017; Barrientos et al. 2008; Risau 1997). Angiogenesis also constitutes a crucial step in cancer development, since it supplies cancerous cells with nutrients for continued growth, creates a system to remove metabolic waste, and facilitates metastasis, which is the invasion of tumour cells into new regions of tissue (Folkman 1995; Byrne 2010; Carmeliet and Jain 2011; Duran et al. 2017; Viallard and Larrivée 2017).

The models we consider in this article describe the dynamics of two cell types involved in angiogenesis: tip and stalk cells (Duran et al. 2017; Betz et al. 2016; Viallard and Larrivée 2017; Carmeliet and Jain 2011; Phng and Gerhardt 2009; Gerhardt et al. 2003; Hellström et al. 2007). Tip cells guide the migration of new sprouts by moving chemotactically up gradients of angiogenic factors (also known as tumour angiogenic factors, or TAFs), which include such molecules as vascular endothelial growth factor (Yadav et al. 2015; Lin et al. 2016). Aside from migrating through the extracellular matrix, tip cells can also branch to create multiple angiogenic sprouts and fuse with other tip or stalk cells, in a process called anastomosis, to create closed loops in the network. Stalk cells, meanwhile, have a more proliferative phenotype than tip cells and are located along the path of tip cell migration. They provide structural support to the new network, and help to establish a lumen through which blood can flow (Duran et al. 2017; Betz et al. 2016; Szymborska and Gerhardt 2018; Iruela-Arispe and Davis 2009).

A variety of continuum and discrete approaches have been used to model tip and stalk cell dynamics; they include (but are not limited to) compartment-based models (Spill et al. 2015), cellular automata (Anderson and Chaplain 1998; Qutub and Popel 2009; Jackson and Zheng 2010; Pillay et al. 2017), the Cellular Potts model (Bauer et al. 2007; Boas et al. 2018), deterministic PDEs (Flegg et al. 2015; Connor et al. 2015), and stochastic differential equations (Bonilla et al. 2014, 2016; Perfahl et al. 2017; Bonilla et al. 2020). They may be implemented in specialised computational frameworks such as Microvessel Chaste or CompuCell3D, to name but two (Grogan et al. 2017; Swat et al. 2009). For a more detailed list of mathematical models for angiogenesis, we refer the interested reader to the following reviews (Scianna et al. 2013; Mantzaris et al. 2004; Heck et al. 2014; Flegg et al. 2020).

The first continuum model that we consider is a coarse-grained system derived from a rule-based ABM in Pillay et al. (2017). The Pillay et al. ABM (henceforth denoted P–ABM) tracks the movement and proliferation of tip and stalk cells in response to a generic TAF on a 2D unit square lattice. Tip cells move via a biased random walk towards increasing TAF concentrations and proliferate to create new sprout branches. Stalk cells are created at sites left vacant when tip cells move, and anastomosis occurs when a tip cell moves into an occupied lattice site.

The coarse-grained PDE describes the P–ABM’s average distribution of tip and stalk cells over time and space. Although the full system of equations is formulated in 2D, it may be reduced to one spatial variable by column averaging the dependent variables, making a mean-field approximation, and non-dimensionalising the equations with appropriate length and time scales. The resulting 1D coarse-grained system can be written in Cartesian coordinates as follows: we denote by *N*(*x*, *t*) and *E*(*x*, *t*) the column averaged tip and stalk cell densities, respectively, at location $x\in [0,L]$ and time $t\in [t_0,\infty )$, with $t_0\ge 0$, and by *C*(*x*, *t*) the column averaged concentration of a generic TAF that regulates the cell dynamics. The system is closed by imposing no-flux boundary conditions for the tip cells (boundary conditions are not required for the stalk cell equation because it is a first-order differential equation with respect to *t* – see below). We assume that the source of TAF is located at $x=L>0$, that there is no TAF at the original vessel where $x = 0$, and that the decay of TAF and its uptake by cells are negligible. Under these assumptions, the coarse-grained model is given by

1 $$\begin{aligned} \frac{\partial N}{\partial t}= & {} \underbrace{\Bigg (D\frac{\partial ^2 N}{\partial x^2}-\chi \frac{\partial }{\partial x}\Big (N\frac{\partial C}{\partial x}\Big )\Bigg )}_{\text {random motion + chemotaxis}}(1-\underbrace{a_nN-a_eE}_{\text {anastomosis}}) + \underbrace{\lambda NC}_{\text {branching}}-\underbrace{\mu a_nN^2-\mu a_e NE}_{\text {anastomosis}}, \nonumber \\ \end{aligned}$$

2 $$\begin{aligned} \frac{\partial E}{\partial t}= & {} \underbrace{\mu N}_{\text {production from tip cell movement}} + \underbrace{a_n \mu N^2 + a_nN\Bigg (D\frac{\partial ^2 N}{\partial x^2}-\chi \frac{\partial }{\partial x}\Big (N\frac{\partial C}{\partial x}\Big )\Bigg )}_{\text {production from tip-to-tip anastomosis}}, \end{aligned}$$

3 $$\begin{aligned} \gamma \frac{\partial C}{\partial t}= & {} \underbrace{\frac{\partial ^2 C}{\partial x^2},}_{\text {diffusion of TAF}} \end{aligned}$$

4 $$\begin{aligned} \begin{aligned}&D\frac{\partial N}{\partial x}-\chi N\frac{\partial C}{\partial x}=0 \ \text {at} \ x = 0,\ L,\\&\ \ \ \ \ \ \ \ C(0,t) = 0, \ C(L,t) = 1,\\&N(x,t_0) = G(x), \ E(x,t_0) = H(x), \end{aligned} \end{aligned}$$

where $D>0$ is the tip cell random motility coefficient, $\chi >0$ represents tip cell sensitivity to TAF, $\lambda >0$ is related to the rate of tip cell branching, $\mu >0$ is related to tip cell movement, and the non-negative functions *G*(*x*) and *H*(*x*) represent the tip and stalk cell densities at time $t_0\ge 0$, respectively. The values of *D*, $\chi$, $\mu$, and $\lambda$ may be written in terms of the P–ABM parameters (Pillay et al. 2017). In Eq. (1), the parameter $0\le a_e\le 1$ is related to the prevalence of tip-to-sprout anastomosis, and in practice is estimated by fitting to data from the P–ABM using a nonlinear least squares method. The parameter $a_n$, by contrast, is a model choice parameter: it either takes the value 0 or 1, depending on whether tip-to-tip anastomosis is included in the microscopic model. For the remainder of this article, we fix $a_n=1$. We refer to Eqs. (1)–(4) as the Pillay PDE system, or P–PDE for short (see Table 1).

In Eq. (1), the expressions representing tip cell movement are multiplied by the nonlinear term $(1-a_nN-a_eE)$, which derives from the volume exclusion rules incorporated in the P–ABM and models the effects of cell elimination due to tip-to-tip and tip-to-sprout anastomosis. This term, however, may cause the P–PDE to become ill-posed if $a_nN+a_eE>1$ (Pillay et al. 2017, 2018). When this occurs, the coarse-grained tip cell equation becomes similar to a negative diffusion model, which is known to yield ill-posed solutions that do not depend continuously on the initial data. Thus, the PDE solutions have the potential to be ill-posed if there are sufficiently large densities of tip and stalk cells. Although in practice multiple signalling cues act to prevent the emergence of such high cell density regions, for instance by creating a “salt-and-pepper” pattern of tip cells as they emerge from a pre-existing vessel (Bentley et al. 2008; Blanco and Gerhardt 2013; Duran et al. 2017), these subcellular processes are outside the scope of this coarse-grained model. Therefore, to ensure well-posedness of the PDE solutions, we simulate the system from $t_0>0$, by which time regions of high tip cell density have been sufficiently eliminated by anastomosis so that $(1-a_nN-a_eE)\ge 0$. Guided by previous numerical implementations from Pillay et al. (2017), we choose $t_0 = 0.2$ with column averaged P–ABM results as initial conditions (see Appendix E for details on the numerical methods).

Equation (3) describes the TAF dynamics.Byrne and Chaplain (1995) determined that for typical parameter values $0<\gamma \ll 1$, hence a quasi-steady state approximation can be made for *C*(*x*, *t*) by setting $\gamma = 0$. The solution to Eq. (3) in this limit, subject to boundary conditions (4), is $C(x,t) \approx C(x) = \nu x$, where $\nu = L^{-1}$. We will assume for the remainder of the article that the TAF concentration is given by this expression.

**Table 1:** The two continuum angiogenesis models investigated in this article

Name of model	Tip and stalk cell evolution equations
Snail-Trail model (ST–PDE)	$\frac{\partial N}{\partial t} =D\frac{\partial ^2 N}{\partial x^2}-\chi \frac{\partial }{\partial x}\Big (N\frac{\partial C}{\partial x}\Big ) + \lambda NC- a_e\mu NE - a_n \mu N^2,$
	$$\frac{\partial E}{\partial t}= \kappa (x)\Big |\frac{\partial N}{\partial x}-\chi N \frac{\partial C}{\partial x}\Big |$$
Pillay PDE model (P–PDE)	$\frac{\partial N}{\partial t}=\Bigg (D\frac{\partial ^2 N}{\partial x^2} -\chi \frac{\partial }{\partial x}\Big (N\frac{\partial C}{\partial x}\Big ) \Bigg )(1-a_nN-a_eE)+\lambda NC-a_e\mu NE-a_n\mu N^2,$
	$$\frac{\partial E}{\partial t} = \mu N + a_n\mu N^2 + a_n N\Bigg (D\frac{\partial ^2 N}{\partial x^2}-\chi \frac{\partial }{\partial x}\Big (N\frac{\partial C}{\partial x}\Big )\Bigg )$$

*N*(*x*, *t*) (tip cell density), *E*(*x*, *t*) (stalk cell density), *C*(*x*, *t*) (TAF concentration, governed by Eq. (3), whose solution is $C(x,t) = x/L = \nu x$). The biological significance of each parameter value is described in the text. All models are closed with the initial and boundary conditions given by Eq. (4). The function $\kappa (x)$ is defined in Eq. (7)

The second continuum angiogenesis model that we investigate is based on the classical “snail-trail” framework. Its defining feature is that the stalk cell density increases at a rate proportional to the magnitude of net tip cell flux, which models the experimental observation that stalk cells proliferate along the path of tip cells (Balding and McElwain 1985; Byrne and Chaplain 1995; Connor et al. 2015). Although originally constructed from a phenomenological scheme proposed by Balding and McElwain (1985), snail-trail models may also be derived from discrete, compartment-based models for angiogenesis (Spill et al. 2015). In these PDE systems, tip cells are assumed to move via chemotaxis up TAF gradients and by random motion. Other processes can also be modelled, such as branching of new tip cells and tip cell elimination by anastomosis (Byrne and Chaplain 1995; Pettet et al. 1996). If *N*(*x*, *t*), *E*(*x*, *t*), *C*(*x*, *t*), *D*, $\chi$, $a_n$, $a_e$, $\mu$, and $\lambda$ have the same physical interpretations as in the P–PDE, then the non-dimensional 1D version of the snail-trail model may be written in Cartesian coordinates as

5 $$\begin{aligned} \frac{\partial N}{\partial t}= & {} \underbrace{D\frac{\partial ^2 N}{\partial x^2}-\chi \frac{\partial }{\partial x}\Big (N\frac{\partial C}{\partial x}\Big )}_{\text {random motion + chemotaxis}}+\underbrace{\lambda NC}_{\text {branching}}-\underbrace{a_n\mu N^2 - a_e\mu NE,}_{\text {anastomosis}} \end{aligned}$$

6 $$\begin{aligned} \frac{\partial E}{\partial t}= & {} \underbrace{\kappa (x)\Big |D\frac{\partial N}{\partial x}-\chi N\frac{\partial C}{\partial x}\Big |.}_{\text {production from net tip cell flux}} \end{aligned}$$

The initial conditions, boundary conditions, and TAF dynamics for this system are given by Eqs. (3)–(4). We again make a quasi-steady state approximation for the TAF dynamics to conclude that the TAF concentration in the snail-trail model is given by $C(x,t) \approx C(x) = \nu x$.

The absolute value sign in Eq. (6) ensures that the stalk cell density is always non-negative, even when the net tip cell flux is negative. In Martinson et al. (2020), we argued that the factor $\kappa (x)$ must be included in the snail-trail framework as a correction for using a net quantity (namely, the net tip cell flux) to estimate the total stalk cell density rate of change. Its derivation, which is reproduced in Appendix F, makes use of the P–ABM rules to obtain the following approximate formula for $\kappa (x)$, which is valid in situations where the TAF gradient is non-zero:

7 $$\begin{aligned} \kappa (x) = \frac{\mu }{\chi \Big |\frac{\partial C}{\partial x}\Big |}. \end{aligned}$$

The corrective factor is a function of *x* because the TAF gradient may vary with respect to space. We have already noted, however, that in this article our column averaged TAF field is $C(x) = \nu x$; since the gradient of this field is constant, this means that $\kappa (x) = \mu /(\chi \nu )$ is also constant. We will refer to Eqs. (5)–(6) as the snail-trail model (ST–PDE, see Table 1).

**Fig. 1:**
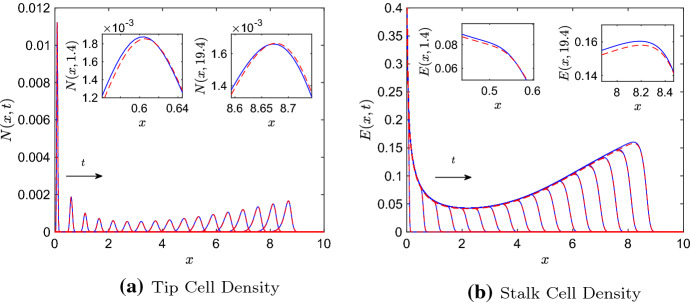
Numerical results of the ST–PDE and P–PDE showing **a** the tip cell density, *N*(*x*, *t*), and **b** the stalk cell density, *E*(*x*, *t*), at times $t = 0.2$, 1.4, $\dots$, 19.4 within the domain $x\in [0,10]$. The insets in both figures show zoomed-in views of the results at (left inset) $t = 1.4$ and (right inset) $t = 19.4$. Key: P–PDE solutions (solid blue lines), ST–PDE solutions (dashed red lines). Parameter values: $D=5\times 10^{-4}$, $\chi = 0.425$, $\mu = 150$, $\lambda = 0.1$, $a_e = 0.05$, $a_n = 1$. The PDEs were initialised at $t = 0.2$ with data from the P–ABM, column averaged in the *y*-direction. For colours, we refer to the online version of this article

Although the P–PDE has a different stalk cell evolution equation than the ST–PDE and contains additional nonlinear terms, Pillay et al. (2017) found that, for certain parameter regimes, numerical solutions to the two continuum models were indistinguishable. We observe this behaviour in Fig. 1, where we present numerical solutions to the ST–PDE and P–PDE. In Fig. 1a, we find that at each time point shown, the largest pointwise difference between solutions is less than 5% of the maximum tip cell density. Similarly, in Fig. 1b the largest pointwise difference between stalk cell solutions is less than 3% of the maximum stalk cell density for each time point shown. Results such as these raise the question of whether solutions to the ST–PDE and P–PDE agree for larger regions of parameter space. Addressing this issue is important, as it would establish conditions under which nonlinear coarse-grained models such as the P–PDE can be represented well by simpler phenomenological systems like the ST–PDE.

### Article outline

In this article, we compare the behaviour of solutions to the ST–PDE and P–PDE in order to determine when they will be in good agreement. In Sect. 2, we use perturbation methods to identify the dominant dynamics of the ST–PDE and P–PDE in parameter regimes that correspond to chemotaxis-dominated tip cell movement and small branching rates. We find that, in such regions of parameter space, both PDE models reduce to the same system at leading order within the domain interior. Numerical simulation confirms that pointwise differences between both solutions are relatively small under the conditions outlined above. In Sect. 3, we construct asymptotic solutions to the leading order systems, which are valid for early times. Numerical simulation demonstrates that such analytic expressions are in good agreement with ST–PDE and P–PDE results. The approach taken in this paper may be used in the future to compare other nonlinear coarse-grained and/or phenomenological models for a given biophysical process.

## Leading order model dynamics

The procedure we use to reduce the ST–PDE and P–PDE to their dominant, leading order dynamics is presented below. For clarity, the details are shown only for the ST–PDE: the analysis for the P–PDE follows *mutatis mutandis*, and is presented in Appendix A. We focus our analysis only on the domain interior: as we have already noted, the continuum models are simulated from $t_0 = 0.2$; at this time point, most of the tip cell mass has moved away from the left-hand boundary into the domain interior. Furthermore, both continuum models represent the migration of cells towards a TAF source, and not the subsequent infiltration of cells into the region producing TAF; this means that both models cease to be valid biological descriptions when the majority of the tip cell mass reaches the right-hand boundary at $x = L$. We therefore restrict our attention to solutions within the domain interior rather than near the boundaries.

We make the following assumptions:

1. All parameter values in the ST–PDE are equal to their counterparts in the P–PDE.
2. The initial and boundary conditions for the ST–PDE and P–PDE are identical.

After substituting equation (7) into the ST–PDE stalk cell evolution equation, we simplify our analysis by reducing the number of parameters in the ST–PDE. We do this by recasting the dependent and independent variables with the following transformations:

8 $$\begin{aligned} u = \frac{a_n\mu }{\lambda }N,\ \ w = \frac{a_e\mu }{\lambda }E,\ \ c = C,\ \ \tau = \lambda t,\ \ X = x \ \sqrt{\frac{\lambda }{D}}, \end{aligned}$$

so that the time scale of interest is $\lambda ^{-1}$ and the length scale is $\sqrt{D/\lambda }$. We write the TAF concentration as a function $c(X,\tau )$ for purposes of clarity, as this allows us to avoid explicitly writing *x* in terms of the new independent variables. Equations (5)–(6) then transform to give

9 $$\begin{aligned} \frac{\partial u}{\partial \tau }= & {} \frac{\partial ^2 u}{\partial X^2}-\frac{1}{\epsilon }\frac{\partial u}{\partial X}+ u(c-u-w), \end{aligned}$$

10 $$\begin{aligned} \frac{\partial w}{\partial \tau }= & {} \beta \Big |\epsilon \frac{\partial u}{\partial X} - u\Big |, \end{aligned}$$

with boundary and initial conditions given by

11 $$\begin{aligned} \epsilon \frac{\partial u}{\partial X}-u= & {} 0 \ \text {at} \ X = 0, \ L\sqrt{\frac{\lambda }{D}}, \end{aligned}$$

12 $$\begin{aligned} u(X,\tau _0)= & {} \frac{a_n}{\alpha }G( X\sqrt{D/\lambda }) =: g(X), \nonumber \\ w(X,\tau _0)= & {} \frac{a_e}{\alpha }H(X\sqrt{D/\lambda }) =: h(X). \end{aligned}$$

In Eqs. (9)–(12), we have introduced the following dimensionless parameter groupings

$$\begin{aligned} \epsilon := \frac{\sqrt{D\lambda }}{\chi \nu }, \ \ \alpha := \frac{\lambda }{\mu }, \ \ \beta := \frac{a_e\mu }{a_n\lambda } = \frac{a_e}{a_n\alpha }. \end{aligned}$$

We remark that, for this specific scenario, $\beta$ is a constant because $\kappa (x)$ and the TAF gradient are constant; in more general scenarios where the TAF gradient varies in space, however, $\beta$ would be a function that also depends on the rescaled spatial variable. Equations (9)–(12) may be compared to the rescaled P–PDE (Eqs. (21)–(22) in Appendix A). Under the following assumption, we may further reduce the ST–PDE using perturbation methods (Bender and Orszag 1999; Hinch 1991; Verhulst 2005):

(A3) Tip cell movement is dominated by chemotaxis and the branching rate is sufficiently small so that $0<\epsilon \ll 1$ and $0<\alpha =\epsilon ^2\varPsi \ll 1$, with $\varPsi \sim O(1)$.

Assumption (A3) is motivated by research that suggests chemotaxis up TAF gradients is the dominant mechanism by which tip cells migrate (Duran et al. 2017; Betz et al. 2016; Bowersox and Sorgente 1982; Carmeliet and Jain 2011). The relationship between $\epsilon$ and $\alpha$ follows from assumption (A1) and the relationship between the coarse-grained model parameters and those of its underlying discrete model (Simpson et al. 2009; Pillay et al. 2017). Namely, if *k* and *h* represent P–ABM parameters related to the response of tip cells to TAF and the spatial step size of the lattice, respectively, then the continuum parameters *D* and $\chi$ may be written as

$$\begin{aligned} D = \frac{\mu h^2}{4}, \ \ \chi = \mu kh^2. \end{aligned}$$

Substituting these expressions into the formula for $\epsilon$, we find that $\epsilon = \sqrt{\alpha }/(2kh\nu ) =\sqrt{\alpha }\varPsi ^{-1/2}$.

We will also assume for the remainder of this section that the ratio $a_e/\alpha$ is bounded in the limit as $\alpha \rightarrow 0$, so that $\beta \sim O(1)$. In practice, this latter assumption may be relaxed: in Appendix B, we show that the leading order dynamics of the ST–PDE and P–PDE are identical for a wider range of values of $\beta$.

With $0<\epsilon \ll 1$, we seek asymptotic solutions for $u(X,\tau )$ and $w(X,\tau )$ of the form

$$\begin{aligned} u(X,\tau )\sim u_0(X,\tau ) + \epsilon u_1(X,\tau ),\ \ \ \ \ w(X,\tau )\sim w_0(X,\tau ) + \epsilon w_1(X,\tau ). \end{aligned}$$

Substitution into Eq. (10), and equating coefficients of $O(\epsilon ^0)$, leads to the following PDE, which describes the dynamics of $w_0(X,\tau )$:

13 $$\begin{aligned} \frac{\partial w_0}{\partial \tau } = \beta |u_0|. \end{aligned}$$

Ideally, the same procedure would be used to identify the leading order dynamics of the tip cell rate Eq. (9); however, this naive approach will fail because the equation contains a term of $O(\epsilon ^{-1})$: in particular, we would find that the leading order tip cell solution gradient is 0, which clearly does not match our observations of the ST–PDE and P–PDE solutions in Fig. 1. We may circumvent this issue by making a change of variables: we let

$$\begin{aligned} y = X - \frac{\tau +X_0}{\epsilon }, \ \ U = u, \ \ W = w, \ \ \widetilde{C} = c, \end{aligned}$$

where $X_0$ is an arbitrary constant that ensures the right boundary location, $y^* = (\lambda L/(\chi \nu ) -\tau -X_0)/\epsilon$, is strictly positive over the time interval of interest. Once again, we have written the steady state TAF concentration as a function $\widetilde{C}(y,\tau )$ for purposes of clarity. We remark that $\widetilde{C}(y,\tau )$ is still bounded between 0 and 1. Substitution of these transformations into Eq. (9) yields

14 $$\begin{aligned} \frac{\partial U}{\partial \tau } = \frac{\partial ^2U}{\partial y^2} + U(\widetilde{C}-U-W), \end{aligned}$$

with boundary conditions

15 $$\begin{aligned} \epsilon \frac{\partial U}{\partial y}-U = 0 \text { at } y=-\frac{\tau }{\epsilon }, \ \frac{\frac{\lambda }{\chi \nu } L-\tau -X_0}{\epsilon }. \end{aligned}$$

We seek a regular perturbation series solution for *U* of the form

$$\begin{aligned} U(y,\tau )\sim U_0(y,\tau ) + \epsilon U_1(y,\tau ). \end{aligned}$$

At leading order, we recover the following PDE describing the dynamics of $U_0$:

16 $$\begin{aligned} \frac{\partial U_0}{\partial \tau } = \frac{\partial ^2U_0}{\partial y^2} + U_0(\widetilde{C}-U_0-W_0), \end{aligned}$$

where the dynamics of $W_0$ are described by Eq. (13) (after a suitable change of variables). The boundary conditions for the leading order solution, found in the limit as $\epsilon \rightarrow 0^+$, are

17 $$\begin{aligned} \lim \limits _{y\rightarrow \pm \infty } U_0(y,\tau ) = 0, \end{aligned}$$

which are homogeneous Dirichlet boundary conditions. We show in Appendix C that tip cell solutions to Eq. (16) with boundary conditions (17) are non-negative, provided the initial condition is also non-negative. We may therefore ignore the absolute value sign in Eq. (13). The resulting system is identical to that obtained from the P–PDE (see Appendix A).

**Fig. 2:**
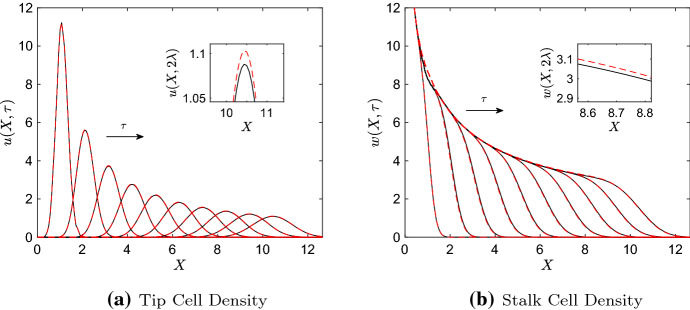
Numerical solution of the leading order dynamics showing how **a** the tip cell density, $u(X,\tau )$, and **b** the stalk cell density, $w(X,\tau )$, evolve over times $\tau = 0.2\lambda$, $0.4\lambda$, $\dots$, $2\lambda$, with $\lambda = 0.16$, along with ST–PDE results (the independent variables of the leading order tip cell solution have been transformed back into the variables *X* and $\tau$ for ease of comparison). The insets in both **a** and **b** show a zoomed-in view of the results at time $\tau = 2\lambda$. Key: ST–PDE solution (solid black lines), leading order solutions (dashed red lines). Parameter values: $D = 10^{-3}$, $\chi = 0.4$, $a_n=1$, $a_e = 0.0391$, $\mu = 160$ (this corresponds to $\epsilon =10^{-3/2}$, $\alpha =10^{-3}$, and $\beta =39.1$). The PDEs were simulated on $\tau \in [0.2\lambda , 2\lambda ]$, $X\in [0, \sqrt{\frac{\lambda }{D}}]$. P–ABM solutions at $\tau =0.2\lambda$, column averaged in the *y*-direction, were used as initial conditions. For colours, we refer to the online article

Figure 2 presents the numerical solution to the leading order system, with the tip cell solution transformed back into the independent variables *X* and $\tau$, along with numerical results from the original ST–PDE. We observe good agreement between both sets of tip cell densities in Fig. 2a: at each time point shown, for instance, the largest pointwise difference between the two solutions is less than 2% of the maximum tip cell density. Similarly, we observe in Fig. 2b that the numerical solution to Eq. (13) is almost indistinguishable from that of the original ST–PDE when the leading order tip cell solution is used to calculate the stalk cell density rate of change. Indeed, at each time point shown the maximum pointwise difference between the two solutions is less than 2% of the maximum stalk cell density. These results support our earlier hypothesis that the leading order dynamics of the ST–PDE within the domain interior are accurately described by Eqs. (13) and (16) when $0<\epsilon \ll 1$ and $0<\alpha \ll 1$.

Crucially, we show in Appendix A that, under assumptions (A1)-(A3) listed above, we may reduce the P–PDE to Eqs. (13) and (16) as well. This result holds regardless of the value of $\beta$, the non-dimensional parameter that depends on the rate of tip-to-sprout anastomosis (see Appendix B for details). In biological terms, this suggests that the ST–PDE and P–PDE are identical to leading order if chemotaxis dominates tip cell movement and if the branching rate is sufficiently small; additionally, the agreement between the two models does not depend on the rate of tip-to-sprout anastomosis. Since the higher-order terms in the tip and stalk cell asymptotic series will be small when $0<\epsilon \ll 1$ and $0<\alpha \ll 1$, this implies that solutions to the original ST–PDE and P–PDE models will be indistinguishable within the domain interior for such parameter regimes.

**Fig. 3:**
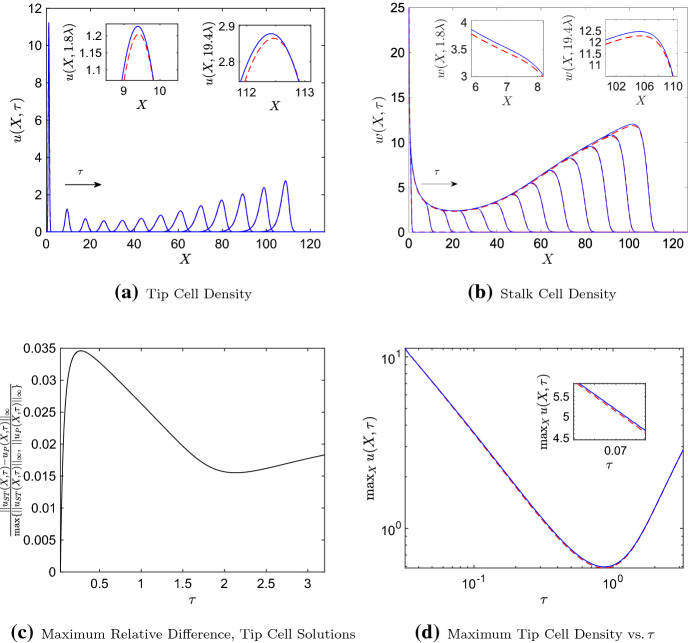
Numerical solutions of the ST–PDE and P–PDE systems showing how **a** the tip cell density, $u(X,\tau )$, and **b** the stalk cell density, $w(X,\tau )$, evolve over times $\tau = 0.2\lambda , 1.8\lambda , ..., 19.4\lambda$, where $\lambda = 0.16$. The insets in **a** and **b** show a zoomed-in view of the solutions at (left inset) $\tau = 1.8\lambda$ and (right inset) $\tau = 19.4\lambda$. The graph in **c** shows the maximum relative difference between tip cell solutions of the ST–PDE, $u_{ST}(X,\tau )$, and P–PDE, $u_{P}(X,\tau )$, against $\tau$. The log-log plot **d** shows how the maximum tip cell density in both models evolves over time $\tau$. The inset in that graph shows a zoomed-in view for small values of $\tau$. Key: ST–PDE (dashed red lines), P–PDE (solid blue lines). Parameter values: $D = 10^{-3}$, $\chi = 0.4$, $a_e = 0.0391$, $a_n = 1$, $\mu = 160$ (this corresponds to $\epsilon =10^{-3/2}$, $\alpha =10^{-3}$, $\beta = 39.1$). The PDEs were simulated on $\tau \in [0.2\lambda ,20\lambda ]$, $X\in [0,10\sqrt{\frac{\lambda }{D}}]$. The P–ABM solutions at $\tau =0.2\lambda$, column averaged in the *y*-direction, were used as initial conditions for the PDE models. For colours, we refer to the online article

We have validated these conclusions with numerical results. Figure 3 presents numerical solutions of the original ST–PDE and P–PDE models listed in Table 1, using a parameter regime for which $0<\epsilon \ll 1$, $0<\alpha \ll 1$, and $\beta \sim O(1)$ (parameter values are listed in the figure caption; see Appendix E for details of the numerical methods). We observe in Fig. 3a that at each time point, the tip cell solution profile resembles a bell curve. We provide a possible explanation for this in Sect. 3, where we show that the leading order tip cell solution has a bell curve profile for early times, regardless of the value of $\beta$. We also see that the amplitude of the tip cell density increases gradually as the solution travels to the right. This most likely occurs because the tip cell branching rate increases near the right-hand boundary (the proliferation rate is proportional to the TAF concentration, which itself is linear with respect to *X*).

The ST–PDE and P–PDE tip cell solutions appear to travel with similar speeds in Fig. 3a. In fact, further investigation shows that the P–PDE speed is within 0.5% of the ST–PDE wave speed, and that in both models the waves accelerate gradually over time (see Appendix E for details on the numerical methods used to arrive at this conclusion).

The ST–PDE tip cell solutions appear to be indistinguishable from those of the P–PDE. We investigated this further by computing the largest pointwise difference between the ST–PDE and P–PDE tip cell solutions over the spatial domain and normalising that value by the maximum tip cell density at each time point (results are presented in Fig. 3c). We observe that the maximum relative pointwise difference between the two sets of solutions lies within the approximate range [0.015, 0.035] (or 1.5%–3.5% of the maximum tip cell density) for the time points shown.

Figure 3b shows the corresponding ST–PDE and P–PDE stalk cell densities for this parameter regime. We see that the P–PDE solution is greater than that of the ST–PDE, even though the solutions to both models appear to have similar profiles. Nevertheless, the size of differences between the ST–PDE and P–PDE solution is small: we calculated the maximum relative pointwise difference between stalk cell solutions using a similar method to the one described above for tip cell solutions (results not shown). In this case, the relative difference between the two sets of stalk cell solutions lies within the approximate range [0.01, 0.02] (1%–2% of the maximum stalk cell density) for the time interval considered here.

In Fig. 3a, the tip cell density initially decreases at a relatively rapid rate. We investigated this behaviour further by creating a log-log plot of the maximum tip cell density against time $\tau$ in Fig. 3d. The graph resembles a straight line until $\tau \approx 0.4$, after which point it begins to curve upwards. In fact, we found that a line with slope $-1.02$ approximates the graph with less than 2% error until $\tau \approx 0.4$ (the equation for the line was computed using least squares regression). We conclude that, for this parameter regime, the maximum tip cell density is approximately proportional to $\tau ^{-1}$ at early times. We explain this observation in Sect. 3, where we find that for this parameter regime there exist self-similar solutions to the leading order ST–PDE and P–PDE dynamics that are initially proportional to $\tau ^{-1}$.

We confirmed that increasing the value of $\epsilon$ in the ST–PDE and P–PDE increases the magnitude of differences between their solutions. We numerically simulated the PDE models for cases in which the random motility coefficient, *D*, was increased from $10^{-3}$ to $10^{-1}$, and in which the chemotactic sensitivity of cells, $\chi$, was decreased from 0.4 to 0.04; in both cases $\epsilon$ increases from about 0.03 to approximately 0.3 but $\alpha$ and $\beta$ are the same as in Fig. 3 (see Figures 1-2 of the Supplementary Material). In both situations, we were able to visually distinguish between the tip and stalk solutions of the two models. Further numerical calculation also revealed that, for such parameter regimes, the relative differences between tip and stalk cell solutions of the two models were larger than the differences shown in Fig. 3.

We have also investigated the case in which the values of $\alpha$ and $\epsilon$ increase simultaneously by changing the value of $\lambda$, the parameter in the ST–PDE and P–PDE related to the branching rate, from $10^{-3}$ to 0.0625 (see Figure 3 of the Supplementary Material). Interestingly, for this parameter regime the differences between the ST–PDE and P–PDE tip/stalk solutions grow noticeably large only over long time periods: the two sets of solutions are indistinguishable from each other at early time points. This apparent discrepancy from our analysis likely arises because the branching rate is proportional to $\lambda$ and to the TAF concentration. Near the left-hand boundary, the TAF concentration is small because it is linear with respect to *X*. In such regions, the overall branching rate remains low even when the value of $\lambda$ increases, and it follows that solutions travelling in such regions are less likely to be affected by changes in $\lambda$. As the tip cell density travels to the right, however, the TAF concentration and branching rate both increase and become more important to solution behaviour. Thus if only the value of $\lambda$ were to increase, then differences between ST–PDE and P–PDE solutions would grow only over long time periods, when the tip cells have invaded regions with sufficiently large TAF concentrations.

Our analysis suggests that $\beta$ does not play an important role at leading order, hence its value is not expected to affect the degree of similarity between the ST–PDE and P–PDE solutions. We confirmed this conclusion via numerical simulation, by increasing the value of $a_e$ in the ST–PDE and P–PDE from 0.0391 to its maximum value of 1 (see Figure 4 of the Supplementary Material; this corresponds to increasing $\beta$ from 39.1 to $10^3$ but keeping $\epsilon$ and $\alpha$ the same values as in Fig. 3).

We have thus found that numerical simulation corroborates the conclusions from our analysis in Sect. 2. Namely, we have found that increasing the value of $\epsilon = \sqrt{D\lambda }/(\chi \nu )$ and/or $\alpha =\lambda /\mu$ increases differences between the ST–PDE and P–PDE solutions. However, changing the value of $\beta$ does not affect whether the two sets of numerical solutions appear indistinguishable from each other. Hence if $0<\epsilon \ll 1$ and $0<\alpha \ll 1$, then solutions for the ST–PDE are in good agreement with those of the P–PDE. This corresponds to biological scenarios in which chemotaxis dominates tip cell movement and sprout branching is rare.

## Self-similar leading order tip cell solutions can exist for small time periods

In this section, we construct asymptotic solutions to the leading order tip cell dynamics derived in Sect. 2. Specifically, we show that Eq. (16), which describes the leading order dynamics of the ST–PDE and P–PDE within the domain interior, admits self-similar solutions for early times (i.e., in the limit as $\tau \rightarrow 0$). The results in Fig. 3d motivate our search for such solutions since we observe that, for early times, the maximum values of the tip cell solutions to the original ST–PDE and P–PDE models are proportional to the same fixed power of $\tau$.

We present the derivation of such self-similar solutions in Appendix D and list the main results of that analysis here. In the case where $\beta \sim O(1)$, for instance, we find that the leading order tip cell solution $U_0(y,\tau )$ may be written in terms of the self-similar expression $\tau ^{-1}\widetilde{U}_0(z)$, where $z = \tau ^{-1/2}(y-\sigma (\tau )\tau )$ and $\sigma (\tau )$ is the wave speed at time $\tau$. Using the principles of dominant balance, we find that in the limit as $\tau \rightarrow 0$ the ordinary differential equation (ODE) describing the dynamics of $\widetilde{U}_0(z)$ is

18 $$\begin{aligned} \frac{d^2\widetilde{U}_0}{dz^2}+\frac{z}{2}\frac{d\widetilde{U}_0}{dz}+\widetilde{U}_0 = \widetilde{U}_0^2, \ \ \ \widetilde{U}_0(\pm \infty ) = 0, \end{aligned}$$

which describes the self-similar tip cell solution profile. The terms that appear in Eq. (18) represent tip-to-tip anastomosis and the movement of tip cells. Terms due to branching and tip-to-sprout anastomosis are proportional to $\tau$ (see Appendix D for details, these correspond to the terms $\widetilde{C}U_0$ and $U_0W_0$ in Eq. (16)), and hence are ignored in the limit as $\tau \rightarrow 0$. Since these terms will become non-negligible for sufficiently large values of $\tau$, we also conclude that this self-similar solution breaks down as $\tau$ increases because it becomes impossible to reduce the dynamics for $\widetilde{U}_0$ to one independent variable. We thus reason that this self-similar solution exists when terms representing branching and tip-to-sprout anastomosis are negligible compared to those describing tip-to-tip anastomosis.

**Fig. 4:**
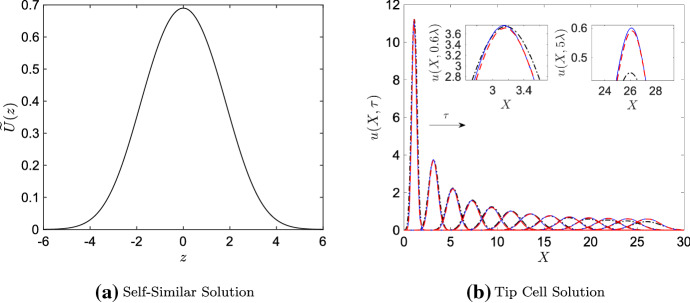
Numerical solution of the boundary value problem given by Eq. (18) in **a** the self-similar variable *z*, and **b** the independent variable *X* for $\tau = 0.2\lambda$, $0.6\lambda$, ..., $5\lambda$, where $\lambda = 0.16$. In **b**, we have plotted the numerical ST–PDE and P–PDE results from Fig. 3 for comparison, and have multiplied the solution to Eq. (18) by $\tau ^{-1}$. In order to better compare the self-similar solution to the ST–PDE and P–PDE results in **b**, we set the maximum value of the self-similar solution at $\tau =0.2\lambda$ equal to that of the ST–PDE and P–PDE tip cell densities at $\tau = 0.2\lambda$. The insets in **b** show a zoomed-in view of the results at (top inset) $\tau = 0.6\lambda$ and (bottom inset) $\tau = 5\lambda$. Key for **b**: P–PDE solutions (solid blue lines), ST–PDE solutions (dashed red lines), solution to Eq. (18) (dashed-dot black line). For colours, we refer to the online article

To our knowledge, there is no closed-form solution to the boundary value problem given by Eq. (18) that is non-trivial (i.e., not $\widetilde{U}_0(z) \equiv 0$). However, a non-trivial numerical solution to the boundary value problem can be computed; it is presented in Fig. 4a. We see that the solution resembles a bell curve, which is consistent with our earlier observations of numerical tip cell solutions. We further compared the similarity solution to results for the ST-PDE and P-PDE by transforming the function in Fig. 4a from an expression in terms of the independent variable *z* into one in terms of the unscaled variable *X*: if we redefine the wave speed as $\phi (\tau ):=1/\epsilon +\sigma (\tau )$, then this transformation is given by

$$\begin{aligned} X = z\sqrt{\tau } + \phi (\tau )\tau + \frac{X_0}{\epsilon }, \end{aligned}$$

which follows from the relationships listed above and in Sect. 2 (see Appendix E for details on the numerical methods used to estimate $\phi (\tau )$ and $X_0$). The transformed self-similar solution is plotted in Fig. 4b over several values of $\tau$ for a parameter regime in which $\epsilon =10^{-3/2}$, $\alpha =10^{-3}$, $\beta =39.1$, and $\lambda =0.16$; the numerical solutions to the original ST–PDE and P–PDE models are also plotted for comparison.

**Table 2:** Dimensionless quantities used to generate the self-similar solution results presented in Fig. 4b. A description of how these values were calculated is in Appendix E

$\epsilon$	$X_0$	$\phi (\tau )$
$10^{-3/2}$	$5.5\times 10^{-5}$	33.45

We observe in Fig. 4b that the self-similar solution is in good agreement with the numerical solutions for the ST–PDE and P–PDE for small values of $\tau$ (the largest pointwise difference between the self-similar solution and the numerical results is on the order of 10% of the maximum tip cell density). As $\tau$ increases, however, the self-similar solution begins to underestimate the ST–PDE and P–PDE solutions. This observation is consistent with our earlier argument that self-similar solutions break down as $\tau$ increases. The results shown in Fig. 4 thus provide evidence that the leading order system admits a similarity solution when $\tau$ is sufficiently small, and that its profile is well described by Eq. (18).

At this point, we have only presented self-similar solutions to the leading order inner tip cell density in the case where $\beta \sim O(1)$. However, similarity solutions also exist for a wider range of values of $\beta$ (this can be shown using a similar procedure to the one presented in Appendix D). When $\beta \ll 1$, for instance, the self-similar solution is identical to the one described above. This makes sense, since we show in Appendix B that terms representing tip-to-sprout anastomosis in the PDEs describing the leading order dynamics are negligible for this parameter regime; thus, this result is consistent with our conclusion that Eq. (18) is an appropriate description of the leading order solution when terms describing branching and tip-to-sprout anastomosis are negligible compared to those for tip-to-tip anastomosis.

When $\beta \gg 1$, however, the self-similar solution is different to the one described by Eq. (18). This is because we find in Appendix B that, for this parameter regime, terms corresponding to tip-to-tip anastomosis are negligible in the leading order tip cell solution. A similar procedure to the one listed in Appendix D demonstrates that in the limit as $\tau \rightarrow 0$, the ODE describing the self-similar solution profile is given by

19 $$\begin{aligned} \frac{d^2\widetilde{U}_0}{dz^2}+\frac{z}{2}\frac{d\widetilde{U}_0}{dz}-A\widetilde{U}_0 = 0, \ \ \ \widetilde{U}_0(\pm \infty ) = 0, \end{aligned}$$

where $z=\tau ^{-1/2}(y-\sigma (\tau )\tau )$ is the same similarity variable as before and *A* is a constant such that $U_0(y,\tau ) = \tau ^{A}\widetilde{U}_0(z)$. The terms in this equation represent tip cell movement only: terms describing tip-to-sprout anastomosis and branching vanish as $\tau \rightarrow 0$. We conclude that the self-similar solution described by Eq. (19) is applicable in cases where terms that represent branching, tip-to-tip anastomosis, and tip-to-sprout anastomosis are negligible compared to those for tip cell movement. The value of *A* is uniquely determined by solving the ODE, satisfying the two boundary conditions, and enforcing the constraint that the solution must be non-negative. This sets $A=-1/2$, and the solution to Eq. (19) is

20 $$\begin{aligned} \widetilde{U}_0(z) = C_1e^{-z^2/4}, \end{aligned}$$

where $C_1$ is a non-negative constant. The solution is a Gaussian function and thus has a bell curve profile. We have verified that it can be in good agreement with the ST–PDE and P–PDE numerical solutions for early times and parameter regimes for which $0<\epsilon \ll 1$, $0<\alpha \ll 1$, and $\beta \gg 1$ (see Figure 5 of the Supplementary Material).

## Discussion and conclusion

In this article, we have analysed two continuum models for angiogenesis: the snail-trail and Pillay PDE systems. The P–PDE is a nonlinear mean-field model that describes the coarse-grained behaviour of a discrete, rule-based ABM (Pillay et al. 2017). By contrast, the snail-trail model is a simpler system constructed from phenomenological arguments (Byrne and Chaplain 1995; Balding and McElwain 1985; Orme and Chaplain 1997; Flegg et al. 2020; Connor et al. 2015). Despite such differences, asymptotic solutions to the two models are identical at leading order within the domain interior under parameter regimes in which chemotaxis dominates tip cell movement and sprout branching is rare. Hence in such biological situations we anticipate that solutions to the ST–PDE will be in good agreement with P–PDE results; we confirmed this with numerical simulation.

In Sect. 3 we demonstrated that the system describing the leading order dynamics of the ST–PDE and P–PDE admits self-similar solutions for early times. Differences in numerical results between the similarity solution, ST–PDE, and P–PDE are relatively small, which indicates that higher order terms in the asymptotic expansions are indeed negligible under parameter regimes in which tip cell movement is dominated by chemotaxis and branching is rare. Our analysis of leading order solutions also provides insight into some observations of the numerical results. For instance, the leading order similarity solution has a bell curve shape regardless of the value of $\beta$, the non-dimensional parameter that is proportional to the rate of tip-to-sprout anastomosis; this may explain why tip cell solutions evolve to such a profile over time.

There are several possible extensions to our work in this article. We limited our search of asymptotic solutions to those only within the domain interior, as we reasoned that they were most relevant to the numerical results given the models and initial conditions. However, this does not exclude the possibility that boundary layers may exist: indeed, numerical results (not shown) reveal that stalk cell solutions of the ST–PDE and P–PDE are different to each other near the left-hand boundary. Thus, one extension of our analysis is to identify potential boundary layers in the leading order ST–PDE and P–PDE solutions, and match them to the dynamics derived in Sect. 2. We also made a quasi-steady state approximation for the TAF dynamics, which simplified our analysis by making the TAF field linear. TAF dynamics may be more complicated in reality, however, and can play a more significant role in the migratory dynamics: cell-induced gradients, for instance, can provide time-dependent guidance cues for tip cells, and will generate spatial heterogeneities in the TAF gradient that were not considered here (Tweedy et al. 2020).

The approach taken in this paper may be generalised to study the relationship between the ST–PDE, P–PDE, and other nonlinear continuum models for angiogenesis. As a particular example, we cite a coarse-grained system from Bonilla et al. (2016), which has been derived from a discrete model that relies on stochastic differential equations to update cell and vessel locations (Bonilla et al. 2014). The continuum model of Bonilla et al. is different from the ST–PDE and P–PDE, but its solutions nevertheless have similar behaviours to the ones investigated in this article: for instance, the leading order tip cell solution in the Bonilla et al. PDE model has been shown to have a profile that resembles a bell curve, and is a travelling wave (Bonilla et al. 2016, 2020). It would be interesting in future work to apply our approach to these other continuum angiogenesis models, as it would provide analytic insight into when solutions from different angiogenesis models can be distinguished from each other, and when simple phenomenological models will be accurate representations of more complicated systems.

Although we identified parameter regimes for which two continuum models are expected to be in good agreement with each other, one open question we did not address in this work is when the continuum models will also closely approximate solutions of discrete models for angiogenesis (which are closer representations of the true biology). Some insight into this question may be gained by considering the derivation of the P–PDE, since this continuum model describes the mean-field behaviour of an ABM and has the same leading order solution as the ST–PDE. In particular, mean-field models such as the P–PDE are appropriate representations of discrete models when spatial correlations between agents can be neglected. In the case of on-lattice ABMs, for instance, this means that the average occupancy of a given lattice site must be independent of the occupancy of its neighbours (Simpson and Baker 2011; Pillay et al. 2017, 2018). However, this mean-field assumption will be violated in most discrete angiogenesis models because of volume exclusion rules due to anastomosis, and the effect of this violation will become more noticeable when many cells are located within a given spatial region (Simpson et al. 2010). We thus expect the P–PDE (and, by extension, the ST–PDE) to be a poor description of discrete angiogenesis results when tip cells branch often.

**Fig. 5:**
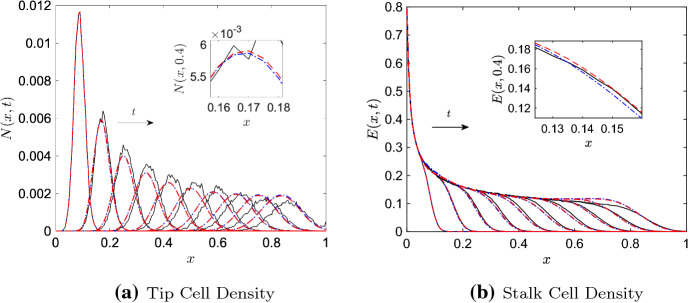
Numerical solutions of the **a** tip cell density, *N*(*x*, *t*), and **b** stalk cell density *E*(*x*, *t*), given by the ST–PDE, P–PDE, and P–ABM at times $t = 0.2$, 0.4, $\dots$, 2. One thousand realisations of the P–ABM, with branching parameter $P_p = 5\times 10^{-2}$, were column averaged in the *y*-direction to generate the results (all other ABM parameter values are the same as those listed in Appendix E). Column averaged P–ABM solutions at $t = 0.2$ were used to initialise the two PDE models, which were simulated on the interval $x\in [0,1]$, $t\in [0.2,2]$. The inset in both panels shows a zoomed-in view of the solution at time $t = 0.4$. Key: column averaged P–ABM distributions (solid black lines), ST–PDE solution (dashed red lines), P–PDE solution (dashed-dot blue lines). PDE parameter values: $D = 10^{-3}$, $\chi = 0.4$, $a_n = 1$, $\mu = 160$. The PDE parameters $\lambda$ and $a_e$ were computed to minimise the squared difference between the PDE solutions and the column averaged ABM data for the time interval shown (see Appendix E for details). For the ST–PDE, we have $\lambda = 0.99$ (95% CI: [0.985, 0.992]), $a_e = 0.0363$ (95% CI: [0.0362, 0.0364]). For the P–PDE, we have $\lambda = 1.115$ (95% CI: [1.111, 1.119]), $a_e = 0.0424$ (95% CI: [0.0423, 0.0425]). For colours, we refer to the online article

Figure 5 presents column averaged P–ABM solutions for a parameter regime in which the branching probability $P_p$ has been increased from $10^{-3}$ to $5\times 10^{-2}$, along with ST–PDE and P–PDE solutions (several continuum parameters have been fitted to the ABM results with a nonlinear least squares method, see the figure caption for their values). We observe that the two continuum models are indistinguishable and hence are in good agreement (the largest pointwise difference between the ST–PDE and P–PDE tip cell solutions at each time point, for example, is less than 4% of the maximum tip cell density). However, the discrepancies between the continuum models and the P–ABM distributions are larger, because we can visually distinguish between the continuum and discrete results. Such results confirm that the continuum models will be increasingly poor descriptions of discrete ABM results when the branching rate increases, even though the two continuum models may still be in good agreement with each other.

Surprisingly, further inspection of Fig. 5 shows that the continuum models do not completely fail at capturing some aspects of the discrete solutions. For example, the shape of the continuum solutions resembles those of the column averaged discrete results, despite the fact that the sizes of their amplitudes are different. Additionally, the continuum models qualitatively capture the experimentally observed brush-border effect, since the tip and stalk cell densities increase as they move closer to the right-hand boundary at $x = 1$. Most importantly, we observe that the leading edge of tip and stalk cell solutions travel with almost identical speeds (they are within 3.5% of each other). We conclude that the continuum models are able to capture some aspects of the underlying discrete solutions, such as the speed of invasion and their qualitative behaviour, even though the calculated densities do not entirely match with the discrete results.

This raises the question of how one should quantify the degree of agreement between discrete and continuum results. For example, “good agreement” could be characterised by sufficiently small absolute pointwise differences between the discrete and continuum solutions, or by small differences between their wave speeds. Clarifying what it means for discrete and continuum models to be in good agreement may provide insight into new metrics that could indicate both the extent of angiogenic progression and when it would be appropriate to apply simple continuum models to study and analyse *in silico* (and eventually *in vivo*) angiogenesis data.

We anticipate that some of our results from this article also hold in a discrete setting due to the relationship between the P–PDE and its underlying ABM. In particular, we anticipate that for parameter regimes in which tip cell branching is rare and cell movement is dominated by chemotaxis, solutions to the two continuum models will be in good agreement with ensemble averages of the P–ABM. If this is true, then this suggests that several summary statistics, such as the number of branches and the average tip cell displacement, could be used to anticipate when discrete solutions would be captured well by continuum models. It would be interesting in future work to evaluate how well such statistics predict the level of similarity between P–ABM and ST–PDE solutions, as this will inform us of when simple phenomenological systems will be good descriptions of more complex discrete angiogenesis data, potentially including those obtained from *in vitro* and *in vivo* approaches.

### Supplementary Information

Below is the link to the electronic supplementary material.

Supplementary material 1 (pdf 20118 KB)

## Figures and Tables

**Fig. 6:**
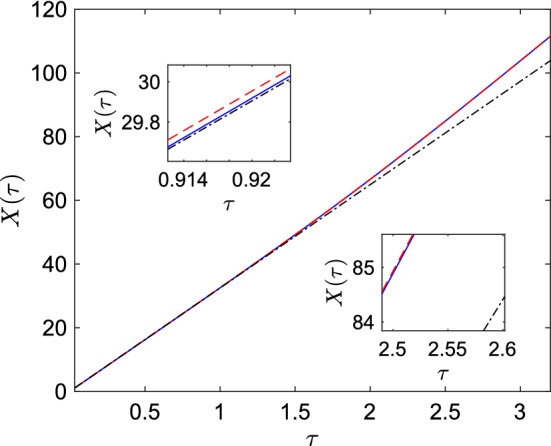
Tip cell density wave front, $X(\tau )$, versus $\tau$ for numerical ST–PDE and P–PDE solutions. These results were generated using the same initial conditions and parameter regime that produced Fig. 3. We have also plotted the line that minimises the squared error with the data for time values within the range $[0.2\lambda , 5\lambda ]$, where $\lambda = 0.16$; its formula is $X(\tau ) = 33.45\tau +0.0546$. The top left inset shows a zoomed-in view of the results for small values of $\tau$, while the bottom right inset shows a zoomed-in view of the results for larger values of $\tau$. Key: ST–PDE wave front (dashed red line), P–PDE wave front (solid blue line), line of best fit to data in the interval $\tau \in [0.2\lambda , 5\lambda ]$ (dashed-dot black line). For colours, we refer to the online article

## Derivation of leading order P–PDE dynamics

Here we derive the leading order dynamics of the P–PDE, using the same procedure and assumptions listed in Sect. 2.

We recast the independent and dependent variables of the P–PDE in terms of the transformations given by Eq. (8), so that *N*(*x*, *t*), *E*(*x*, *t*), and *C*(*x*, *t*) map to the functions $u(X,\tau )$, $w(X,\tau )$, and $c(X,\tau )$, respectively. This leads to the equations

21 $$\begin{aligned} \frac{\partial u}{\partial \tau }= & {} \Big (\frac{\partial ^2 u}{\partial X^2}-\frac{1}{\epsilon }\frac{\partial u}{\partial X}\Big )\Big (1-\alpha (u+w)\Big ) + u(c-u-w), \end{aligned}$$

22 $$\begin{aligned} \frac{\partial w}{\partial \tau }= & {} \beta \Big [u + \alpha u^2 + \alpha ^2u\Big (\frac{\partial ^2 u}{\partial X^2}-\frac{1}{\epsilon }\frac{\partial u}{\partial X}\Big )\Big ], \end{aligned}$$

which are subject to the initial and boundary conditions given by Eqs. (11)–(12) in Sect. 2. Under assumption (A3) of Sect. 2, we let $0<\epsilon \ll 1$, $0<\alpha =\epsilon ^2\varPsi \ll 1$, where $\varPsi$ is a constant of *O*(1). We may thus further reduce the P–PDE using perturbation methods. We will assume for the remainder of this appendix section that $\beta \sim O(1)$ (we show in Appendix B that the leading order dynamics are the same for the ST–PDE and P–PDE, even for different values of $\beta$).

Using the same procedure as outlined in Sect. 2, we find that the dynamics of the leading order stalk cell solution, $w_0(X,\tau )$, are given by

23 $$\begin{aligned} \frac{\partial w_0}{\partial \tau } = \beta u_0. \end{aligned}$$

The dynamics of the P–PDE leading order tip cell solution are found by transforming the independent and dependent variables in Eq. (21) as

$$\begin{aligned} y = X-\frac{\tau +X_0}{\epsilon } \ \ \ U = u, \ \ \ W = w, \ \ \ \widetilde{C} = c, \end{aligned}$$

where $X_0$ is an arbitrary constant, so that $u(X,\tau )$, $w(X, \tau )$, and $c(X,\tau )$ map to functions $U(y,\tau )$, $W(y,\tau )$, and $\widetilde{C}(y,\tau )$. This leads to the equation

24 $$\begin{aligned} \frac{\partial U}{\partial \tau } = \frac{\partial ^2 U}{\partial y^2}-\epsilon \varPsi \Big (\epsilon \frac{\partial ^2 U}{\partial y^2}-\frac{\partial U}{\partial y}\Big )\Big (U+W\Big ) + U(\widetilde{C}-U-W), \end{aligned}$$

We substitute the perturbation series

$$\begin{aligned} U(y,\tau )\sim U_0(y,\tau ) + \epsilon U_1(y,\tau ), \end{aligned}$$

and identify the leading order dynamics by equating terms of $O(\epsilon ^0)$. This leads to the leading order P–PDE tip cell equation

25 $$\begin{aligned} \frac{\partial U_0}{\partial \tau } = \frac{\partial ^2 U_0}{\partial y^2} + U_0(\widetilde{C}-U_0-W_0), \end{aligned}$$

where the dynamics describing the leading order stalk cell solution, $W_0(y,\tau )$, are given by Eq. (23) (after a suitable transformation of variables). Crucially, the resulting inner system is equivalent to that of the ST–PDE in Sect. 2, since we show in Appendix C that the leading order tip cell solutions to the ST–PDE are non-negative. We have confirmed that numerical solutions to the above inner system are in good agreement with results from the full P–PDE (see Figure 6 of the Supplementary Material).

## Leading order dynamics for different values of $\beta$

In Sect. 2 and Appendix A, we assumed $\beta \sim O(1)$ when proving that the ST–PDE and P–PDE are identical at leading order. We may, however, relax this condition: this section presents the procedure for reducing the ST–PDE to leading order for the cases in which $\beta \gg 1$ and $\beta \ll 1$ (the analysis for the P–PDE follows naturally, and is omitted for clarity).

**Table 3:** Outer and inner leading order dynamics of the ST–PDE and P–PDE for all positive values of $\beta$

	Leading order stalk cell dynamics	Leading order tip cell dynamics $\Big (y = X-\frac{\tau +X_0}{\epsilon },\ X_0\in \mathbb {R}\Big )$
${\varvec{\beta }}\sim O(1)$	$\frac{\partial w_0}{\partial \tau }=\beta u_0$	$\frac{\partial U_0}{\partial \tau } = \frac{\partial ^2U_0}{\partial y^2} + U_0(\widetilde{C}-U_0-W_0)$
${\varvec{\beta }}\gg {\varvec{1}}$	$\frac{\partial w_0}{\partial \tau }={\widetilde{\beta }} {\bar{u}}_0$	$\frac{\partial {\bar{U}}_0}{\partial \tau } = \frac{\partial ^2 {\bar{U}}_0}{\partial y^2}+ {\bar{U}}_0(\widetilde{C}-W_0)$
(${\widetilde{\beta }}=\beta \epsilon ^{m}$, $m>0$)		
${\varvec{\beta }}\ll {\varvec{1}}$	$\frac{\partial {\bar{w}}_0}{\partial \tau }={\widehat{\beta }} u_0$	$\frac{\partial U_0}{\partial \tau } = \frac{\partial ^2 U_0}{\partial y^2}+ U_0(\widetilde{C}-U_0)$
(${\widehat{\beta }}=\beta \epsilon ^{-m}$, $m>0$)		

In all cases, $u(X,\tau )$ and $U(y,\tau )$ represent the tip cell density, $w(X,\tau )$ and $W(y,\tau )$ the stalk cell density, and $c(X,\tau )$ and $\widetilde{C}(y,\tau )$ the TAF concentration. We refer to Sect. 2 and Appendices A and B for the derivation of these systems

When $\beta \gg 1$, we may assume that $\beta = {\widetilde{\beta }}\epsilon ^{-m}$, where $m>1$ and ${\widetilde{\beta }}$ is a constant of *O*(1). To derive the leading order dynamics, we rescale the dependent variable *u* so that $u = \epsilon ^m{\bar{u}}$ and $u(X,\tau )\mapsto {\bar{u}}(X,\tau )$. Substitution into Eqs. (9)–(10) yields

26 $$\begin{aligned} \frac{\partial {\bar{u}}}{\partial \tau }= & {} \frac{\partial ^2 {\bar{u}}}{\partial X^2}-\frac{1}{\epsilon }\frac{\partial {\bar{u}}}{\partial X}+ {\bar{u}}(c-\epsilon ^m{\bar{u}}-w), \end{aligned}$$

27 $$\begin{aligned} \frac{\partial w}{\partial \tau }= & {} {\widetilde{\beta }}\big |\epsilon \frac{\partial {\bar{u}}}{\partial X} - {\bar{u}}\big |. \end{aligned}$$

From this point, the procedure for deriving the outer and inner systems is the same as in Sect. 2. We find that the dynamics governing the leading order stalk cell density, $w_0(X,\tau )$, are given by

28 $$\begin{aligned} \frac{\partial w_0}{\partial \tau } = {\widetilde{\beta }}|{\bar{u}}_0|. \end{aligned}$$

Similarly, the leading order tip cell dynamics, ${\bar{U}}_0(y,\tau )$, may be identified using the same transformations presented in Sect. 2. They are given by

29 $$\begin{aligned} \frac{\partial {\bar{U}}_0}{\partial \tau } = \frac{\partial ^2 {\bar{U}}_0}{\partial y^2}+ {\bar{U}}_0(\widetilde{C}-W_0), \end{aligned}$$

where the dynamics of $W_0(y,\tau )$ are given (after a suitable transformation of variables) by Eq. (28). The boundary conditions are given at leading order by

30 $$\begin{aligned} \lim \limits _{y\rightarrow \pm \infty }{\bar{U}}_0(y,\tau ) = 0. \end{aligned}$$

A similar proof to the one in Appendix C shows that the leading order tip cell solution is non-negative, provided its initial condition is non-negative. Therefore we may drop the absolute value sign in Eq. (28). Crucially, the resulting leading order system is identical to the one derived for the P–PDE.

For the case in which $\beta \ll 1$, we rewrite the parameter in terms of a power of $\epsilon$, such that $\beta = {\widehat{\beta }}\epsilon ^m$, where $m>0$ and ${\widehat{\beta }}$ is a constant of *O*(1). We rescale the dependent variable *w* in the ST–PDE, such that $w = \epsilon ^m{\bar{w}}$ and $w(X,\tau )\mapsto {\bar{w}}(X,\tau )$. Substitution into Eqs. (9)–(10) leads to the system

31 $$\begin{aligned} \frac{\partial u}{\partial \tau }= & {} \frac{\partial ^2 u}{\partial X^2}-\frac{1}{\epsilon }\frac{\partial u}{\partial X}+ u(c-u-\epsilon ^m{\bar{w}}), \end{aligned}$$

32 $$\begin{aligned} \frac{\partial {\bar{w}}}{\partial \tau }= & {} {\widehat{\beta }}\Big |\epsilon \frac{\partial u}{\partial X} - u\Big |. \end{aligned}$$

We deduce that the dynamics of the leading order stalk cell solution, ${\bar{w}}_0(X,\tau )$, are given by

33 $$\begin{aligned} \frac{\partial {\bar{w}}_0}{\partial \tau } = {\widehat{\beta }} |u_0|, \end{aligned}$$

while the dynamics of the leading order tip cell solution are given by

34 $$\begin{aligned} \frac{\partial U_0}{\partial \tau } = \frac{\partial ^2 U_0}{\partial y^2}+ U_0(\widetilde{C}-U_0), \end{aligned}$$

where $y = X-\frac{\tau +X_0}{\epsilon }$, subject to the homogeneous Dirichlet boundary conditions

35 $$\begin{aligned} \lim \limits _{y\rightarrow \pm \infty } U_0(y,\tau )=0. \end{aligned}$$

If the initial condition for the leading order tip cell solutions is non-negative, then a similar proof to the one outlined in Appendix C shows that their solutions are non-negative at all other times. Thus we may neglect the absolute value signs in the leading order stalk cell evolution equation. The resulting inner and outer systems are identical to those given by the P–PDE.

Table 3 summarises all of the possible outer and inner systems for the ST–PDE and P–PDE when $0<\epsilon \ll 1$ and $0<\alpha \ll 1$.

## Leading order ST–PDE solutions are non-negative

We show in this appendix that the solution to the leading order ST–PDE tip cell dynamics given by Eq. (16) are non-negative, provided the initial condition is also non-negative. We will assume that solutions are at least twice differentiable.

For sake of contradiction, assume that there exists a point $y^*\in \mathbb {R}$ such that at time $\tau _1>0$, $U_0(y^*,\tau _1) < 0$. Since the PDE solution is twice differentiable, it is continuous, so there must have existed some time $0\le \tau ^*<\tau _1$ such that $U_0(y^*,\tau ^*) = 0$ and $\frac{\partial U_0}{\partial \tau }(y^*,\tau ^*)<0$. We also conclude that $U_0(y,\tau ^*)\ge 0$ for all other values of *y*, since $y^*$ is the first point at which the solution becomes negative.

Using Taylor’s theorem, the second derivative in Eq. (16) may be written as

$$\begin{aligned} \frac{\partial ^2 U_0}{\partial y^2}(y^*,\tau ^*)=\lim \limits _{\varDelta y\rightarrow 0}\frac{U_0(y^*+\varDelta y,\tau ^*)-2U_0(y^*,\tau ^*)+U_0(y^*-\varDelta y,\tau ^*)}{\varDelta y^2}, \end{aligned}$$

which is well-defined because $U_0(y,\tau )$ is twice differentiable. Since $U_0(y^*,\tau ^*) = 0$ and $U_0(y\pm \varDelta y,\tau ^*)\ge 0$, the second derivative must be non-negative. Since the nonlinear reaction term of Eq. (16) is equal to 0 when $U_0 = 0$, this means that $\partial U_0/\partial \tau \ge 0$ at $(y^*,\tau ^*)$, which is a contradiction. We conclude that any twice differentiable solution to Eq. (16) with smooth, non-negative initial data is also non-negative for $\tau \ge 0$.

## Derivation of self-similar tip cell solutions

In this appendix, we show that the leading order tip cell solution is self-similar in the limit as $\tau \rightarrow 0$. We focus on the case for which $\beta \sim O(1)$; a similar procedure can be used to derive self-similar solutions for a wider range of values for $\beta$, using the inner leading order dynamics derived in Appendix B.

In addition to the assumptions made in Sect. 2, we will simplify our analysis by assuming that the following are true:

1. The leading order tip cell solution dynamics are given by Eq. (16) with boundary conditions (17).
2. The TAF concentration $\widetilde{C}(y,\tau )$ is bounded between 0 and 1, as is the case in Sect. 2.
3. The initial leading order tip and stalk cell densities are non-negative and bounded.
4. The wave speed of leading order tip cell solutions, $\sigma (\tau )$, may depend on time but is bounded such that $\sigma (\tau )\tau$ and $\sigma '(\tau )\tau \rightarrow 0$ as $\tau \rightarrow 0$. This assumption is inspired by our numerical results, which suggest that solutions to the ST–PDE and P–PDE travel short distances in small time periods but have wave speeds that vary over time (see Fig. 6).

We seek self-similar solutions by substituting into Eq. (16) the expression

$$\begin{aligned} U_0(y,\tau ) =\tau ^A\widetilde{U}_0\big (\tau ^B(y-\sigma (\tau )\tau )\big ) = \tau ^A\widetilde{U}_0(z), \end{aligned}$$

where *A* and *B* are constants to be determined, $\sigma (\tau )$ is the tip cell wave speed at time $\tau$, and $z=\tau ^B(y-\sigma (\tau )\tau )$ is the self-similar variable. We recast the equation in terms of the independent variables *z* and $\tau$. To avoid explicitly writing the TAF concentration as a function of *z*, we write $\widetilde{C}(y,\tau )$ as $\widetilde{c}(z,\tau )$. Similarly, we use $\widetilde{W}_0$ to denote the leading order stalk cell density in terms of *z* and $\tau$. These substitutions lead to the equation

36 $$\begin{aligned} A\widetilde{U}_0+\Bigg (Bz-\tau ^{1+B}\Big (\sigma (\tau )+\tau \frac{d\sigma }{d\tau }\Big )\Bigg )\frac{d\widetilde{U}_0}{dz} = \tau ^{1+2B}\frac{d^2\widetilde{U}_0}{dz^2} + \Big (\tau \widetilde{c}-\tau \widetilde{W}_0-\tau ^{1+A}\widetilde{U}_0\Big )\widetilde{U}_0. \end{aligned}$$

The values of *A* and *B* are chosen to eliminate as many powers of $\tau$ in Eq. (36) as possible, while simultaneously ensuring that no term blows up to infinity as $\tau \rightarrow 0$. This uniquely sets $A = -1$ and $B=-1/2$. However, the resulting equation does not describe self-similar solutions in general. We conclude that self-similar solutions do not exist for sufficiently large values of $\tau$. However, in the limit as $\tau \rightarrow 0$ one can recover an ODE for $\widetilde{U}$ as a function of *z*. This is because the TAF concentration, wave speed, and stalk cell densities are all bounded: since such quantities are multiplied by positive powers of $\tau$ in Eq. (36), they vanish when $\tau \rightarrow 0$. Such simplifications lead to the ODE (18).

## Numerical methods

Initial conditions for the ST–PDE and P–PDE were constructed from ensemble averages of the P–ABM, which were generated using the following algorithm from Pillay et al. (2017). ABM parameter values are listed in Table 4.

Choose $\varDelta t$, the time step, and $t_{\text {final}}$, the time at which to terminate the ABM solution. Set $t=0$.

Place tip cells at (*x*, *y*) = (0, 2*h*), (0, 4*h*), ..., (0, 1).

While $t < t_{\text {final}}$ and tip cells (TCs) have not reached the right edge of the lattice at

$$x = L:$$

1. Choose $N_{TC}$ tip cells at random with replacement, where $N_{TC}$ is the current number of tip cells.
  **Loop 1**: For 1 to $N_{TC}$
  - Choose a random number $r_1\in [0,1]$.
  - If $r_1\le P_m$, the tip cell moves:
    (i) Choose a random number $r_2\in [0, 1]$.
    (ii) A stalk cell is placed at the lattice site and the tip cell moves left/right/up/down *h* units according to the probabilities $P_{x^{\pm }} = 0.25\times [1\pm k(c(x_i+h, y_j)-c(x_i-h, y_j))]$, $P_{y^{\pm }} = 0.25\times 1\pm [k(c(x_i, y_j+h)-c(x_i, y_j-h))]$, where $c(x_i,y_j)$ is the TAF concentration at location $(x_i,y_j)$.
    (iii) If the tip cell moves into a site occupied by one (or more) tip cells, then tip-to-tip anastomosis occurs and the tip cells are removed.
    (iv) Otherwise, if tip cell moves into a site occupied by a stalk cell that does not belong to the same sprout, then tip-to-sprout anastomosis occurs and the tip cell is removed.
    v) If anastomosis does not occur, then tip cell moves into the target site.
  End Loop 1
2. Choose $N_{TC}$ tip cells at random with replacement, where $N_{TC}$ is the number of tip cells after completing Loop 1.
  **Loop 2**: For 1 to $N_{TC}$
  - Choose a random number $r_3\in [0, 1]$. Define $P_b := P_p\times c(x_i,y_j)$, where $c(x_i,y_j)$ is the TAF concentration at the tip cell’s location.
  - If $r_3\le P_b$, then branching occurs.
  End Loop 2
3. Set time $$t = t + \varDelta t$$.

End While Loop

**Table 4:** P–ABM parameter values used to generate initial conditions for the ST–PDE and P–PDE

$t_{\text {final}}$	$P_m$	*h*	*k*	$P_p$	$\varDelta t$
2	1	$200^{-1}$	100	$10^{-3}$	$160^{-1}$

One thousand realisations of the P–ABM were column-averaged in the *y*-direction at $t = 0.2$ to set up initial conditions for the PDE models. We initialised the PDEs at time $t = 0.2$, rather than $t = 0$, as previous investigations have shown that the P–PDE can become ill-posed if it is initialised at earlier times (Pillay et al. 2017, 2018).

The 1D PDE models were solved using the method of lines (Schiesser 1991). Using grid points with spatial step size $\varDelta x = 1/200$, spatial derivatives were discretised using central finite difference formulae. The systems were solved with MATLAB’s ode15s solver, which implements adaptive time-stepping for stiff ODE problems (Shampine and Reichelt 1997).

In Sects. 2 and 3, we transformed solutions of the ST–PDE and P–PDE from functions of the form *N*(*x*, *t*) and *E*(*x*, *t*) to $u(X,\tau )$ and $w(X,\tau )$, respectively, so as to facilitate comparison with our analysis. This is done using the formulae listed in Eq. (8).

The solution to Eq. (18), presented in Fig. 4a, was computed numerically using the MATLAB library Chebfun, which applies spectral methods and Newton’s method to solve nonlinear boundary value problems (Driscoll et al. 2014). To approximate the infinite domain, the solution was computed on the interval $z\in [-100, 100]$, which is two orders of magnitude larger than the region over which the solution gradient varies the most. The numerical solution was computed using the initial guess $\widetilde{U_0}(z)=\exp (-z^2)$.

The quantities $X_0$ and $\phi (\tau )$, which are used to transform the self-similar solution into the independent variable *X* in Fig. 4b, are calculated in the following manner: the wave speed $\phi (\tau )$ is taken to be the derivative of $X(\tau )$, a function that describes the tip cell wave front location at time $\tau$. We define the wave front location as the mean location of the tip cell solutions:

37 $$\begin{aligned} X(\tau ) := \frac{\int _{-\infty }^{\infty } Xu(X,\tau )dX}{\int _{-\infty }^{\infty } u(X,\tau )dX}. \end{aligned}$$

In the case where $X(\tau )$ is approximately linear, $\phi (\tau )$ is estimated as the slope of the line that minimises the squared error with the data of $X(\tau )$ versus $\tau$, and the value of $X_0$ is the *y*-intercept of this line of best fit.

Figure 6 shows a plot of $X(\tau )$ against $\tau$ for the same parameter regime and initial conditions listed in Fig. 3. We observe that the ST–PDE and P–PDE models have roughly identical wave speeds for the time points considered here, and that the ST–PDE and P–PDE tip cell solutions gradually accelerate over time. For early times, however, the two solutions appear to travel with constant speed: we confirmed this by plotting a straight line that minimises the squared error with the numerical solutions (the line was fit to data in the range $\tau \in [0.2\lambda , 5\lambda ]$, where $\lambda = 0.16$). We see in Fig. 6 that the line of best fit is a good approximation to the numerical results for sufficiently small values of $\tau$ (for $\tau <0.8$, the $R^2$-value is greater than 0.99). We conclude that for $\tau \approx 0$, the wave speed $\phi (\tau )$ is approximately constant; its value is given in Table 2 of Sect. 3.

## Derivation of snail-trail model corrective factor

In this appendix, we provide a brief overview of our derivation of $\kappa (x)$, the corrective factor in the snail-trail model given by Eqs. (5)–(6). For more details on the derivation, we refer to the original article by Martinson et al. (2020).

We let $\widetilde{\kappa }$ be a non-dimensional factor in the snail-trail model that corrects for neglected vessel production in directions other than that of the migrating front. Since a new vessel is itself made up of stalk cells, ${\widetilde{\kappa }}$ is related to $\kappa$ through the relationship $\kappa ={\widetilde{\kappa }}/h_{EC}$, where $h_{EC}$ represents the length of a typical stalk cell. Either factor must be included in the snail-trail modelling framework because the tip cell flux, a net quantity, tends to underestimate the total vessel/stalk cell density rate of change. Thus, if $\text {J}_{net}$ measures the average net tip cell flux in the *x*-direction within a time step $\varDelta t$ and spatial interval *h*, then ${\widetilde{\kappa }}|\text {J}_{net}|$ is equal to the (true) density of new vessels that are created on average within this interval (note that we have absorbed any dependence of *h* and $\varDelta t$ into ${\widetilde{\kappa }}$). In order to derive a function for ${\widetilde{\kappa }}$ (and, by extension, $\kappa$), we examine how the above statement translates into a discrete setting.

We consider the P–ABM framework as a discrete representation of tip and stalk cell movement following a snail-trail assumption. In the P–ABM, tip cell movement is a 2D biased random walk towards increasing concentrations of TAF. The ABM is assumed to have *N* lattice sites that are equally spaced with step size *h*, where *h* represents the length of a typical cell. We assume, for sake of simplicity, that a tip cell length is equivalent to that of a stalk cell (so $h_{EC}$ = *h*). Just as for the continuous snail-trail model, we assume that the TAF concentration *C*(*x*) is prescribed and independent of time *t* and the spatial variable *y*.

Within a time step $\varDelta t$, tip cells are chosen to move with a constant probability $P_m$. Once chosen to move, the direction in which a tip cell travels is selected according to the following probabilities: if we denote the probability of moving left as $P_{x^-}$ and right as $P_{x^+}$, then these values are defined at $x_i\in (0,1)$ as

38 $$\begin{aligned} P_{x^\pm } = \frac{1\pm g_x(x_i)}{4}, \end{aligned}$$

where for $$0<i<(N-1)$$,

39 $$\begin{aligned} g_x(x_i) := k\big (C(x_{i}+h)-C(x_{i}-h)\big ). \end{aligned}$$

For example, if $g_x = -1/2$, then $P_{x^-}=3/8$ and $P_{x^+} = 1/8$ (i.e., the tip cell is more likely to move in the left direction). Similar formulae are used to determine the movement of tip cells in the *y*-direction. The parameter value *k* in Eq. (39) ensures that no numerator in Eq. (38) can become negative (so that $|g_x(x)|\le 1$); the value of *k* does not vary with respect to location. In the P–ABM, a discrete version of the snail-trail assumption is incorporated by having stalk cells proliferate in the space left empty by a moving tip cell. In other words, any new vessel production within a time step $\varDelta t$ is equal to the number of times tip cells move, since stalk cells will proliferate to occupy the resulting empty space.

This leads us to immediately identify a discrete analogue of $|\text {J}_{net}|$, the magnitude of the tip cell net flux in the *x*-direction: it is simply the expected net number of jumps that tip cells make from a lattice site in the *x*-direction (we use expected values here because of the stochastic nature of the discrete model). We may then interpret the value of $\kappa$ as the total amount of new vessel density produced for every unit of net tip cell movement in the *x*-direction.

We now use the rules of the P–ABM to compute the expected number of jumps that tip cells make in the *x*-direction. We define $X_R$ and $X_L$, to be random variables that measure the total number of rightward and leftward jumps that originate from lattice point $x_i$, respectively. Since cells may only move in these two directions, we model these random variables using multinomial distributions. We also define the random variable $X_{net}=X_R-X_L$, which measures the net number of rightward jumps made from lattice site $(x_i, y_j)$. By the argument above, the net tip cell flux in the *x*-direction is defined by $\text {J}_{net} :=\mathbb {E}[X_{net}]$. Its magnitude is given as $|\text {J}_{net}|$.

Since we have reasoned that ${\widetilde{\kappa }}|\text {J}_{net}|$ is the total vessel density produced (or, equivalently, total jumps that occur in the *x*-direction) from a given lattice site within the time step $\varDelta t$ and spatial interval *h*, it follows that when the value of $|\text {J}_{net}|$ is normalised to 1, ${\widetilde{\kappa }}$ becomes equal to the total number of jumps in the *x*-direction from lattice site $(x_i, y_j)$. We exploit this information to calculate the expected values of our random variables: for example, the probability of executing *m* rightward jumps becomes

40 $$\begin{aligned} P(X_R=m) = \left( {\begin{array}{c}{\widetilde{\kappa }}\\ m\end{array}}\right) (P_{x^+})^m(1-P_{x^+})^{{\widetilde{\kappa }}-m}, \end{aligned}$$

where $$\left( {\begin{array}{c}{\widetilde{\kappa }}\\ m\end{array}}\right) $$ is the binomial coefficient. A similar equation holds for $X_L$. The expected values of $X_R$ and $X_L$ are thus given by

41 $$\begin{aligned} \mathbb {E}[X_R] = {\widetilde{\kappa }} P_{x^+}, \ \ \mathbb {E}[X_L] = {\widetilde{\kappa }} P_{x^-}, \end{aligned}$$

when $|\text {J}_{net}|$ is normalised to 1, so that

$$\begin{aligned} \mathbb {E}[X_{net}] = \mathbb {E}[X_R]-\mathbb {E}[X_L] = {\widetilde{\kappa }}(P_{x^+}-P_{x^-}). \end{aligned}$$

Since Eqs. (40)–(41) are applicable when $|\text {J}_{net}|=1$, we use Eq. (38) to deduce that

$$\begin{aligned} |\text {J}_{net}|= |\mathbb {E}[X_{net}]|= \frac{{\widetilde{\kappa }}}{2} |g_x| = 1, \end{aligned}$$

with $g_x$ defined by Eq. (39). Hence

42 $$\begin{aligned} {\widetilde{\kappa }} = \frac{2}{|g_x|}. \end{aligned}$$

From this equation, it is clear that $\kappa$ is non-negative.

We may apply Taylor’s theorem to simplify Eq. (42): assuming that the average cell length is sufficiently small so that $0<h\ll 1$, we may write $g_x$ as

$$\begin{aligned} g_x(x) = k(C(x+h)-C(x-h)) = k\Big (2h\frac{\partial C}{\partial x} + O(h^3)\Big )\approx 2kh\frac{\partial C}{\partial x}(x). \end{aligned}$$

Substitution of this expression into Eq. (42) yields

43 $$\begin{aligned} {\widetilde{\kappa }}(x) \approx \frac{1}{kh\Big |\frac{\partial C}{\partial x}\Big |}, \end{aligned}$$

hence ${\widetilde{\kappa }}(x)$ is inversely proportional to the magnitude of the local TAF gradient.

It is possible to further transform Eq. (43) so that $\widetilde{\kappa }(x)$ is in terms of the continuum parameters *D* and $\chi$. This simplification follows from the relationship between biased random walk models and advection-diffusion equations (Simpson et al. 2009; Pillay et al. 2017). Namely, if $P_m$ denotes the probability that a tip cell moves within a time step $\varDelta t$, then the relationship between the discrete and continuum parameters is

44 $$\begin{aligned} D= \lim \limits _{h\rightarrow 0,\varDelta t\rightarrow 0}\frac{P_m h^2}{4\varDelta t}, \ \ \chi = \lim \limits _{h\rightarrow 0,\varDelta t\rightarrow 0}\frac{P_m kh^2}{\varDelta t}, \end{aligned}$$

under the assumption that the above limits exist, are non-zero, and are finite. Substituting Eq. (44) into Eq. (43) yields

45 $$\begin{aligned} {\widetilde{\kappa }}(x) = \frac{4D}{h\chi \Big |\frac{\partial C}{\partial x}\Big |} = \frac{\mu h}{\chi \Big |\frac{\partial C}{\partial x}\Big |}, \end{aligned}$$

where we have defined $\mu := P_m/\varDelta t$. From here, it is straightforward to derive Eq. (7) by using the relationship $\kappa (x) = {\widetilde{\kappa }}(x)/h_{EC}={\widetilde{\kappa }}(x)/h$.
