# Supplementary material for: Comparative analysis of continuum angiogenesis models
Source: J Math Biol. 2021 Feb 23;82(4):21. doi: 10.1007/s00285-021-01570-w (PMC7900093; doi:10.1007/s00285-021-01570-w)
Supplement: Supplementary file 1 — Supplementary material 1 (pdf 20118 KB) [file 285_2021_1570_MOESM1_ESM.pdf]

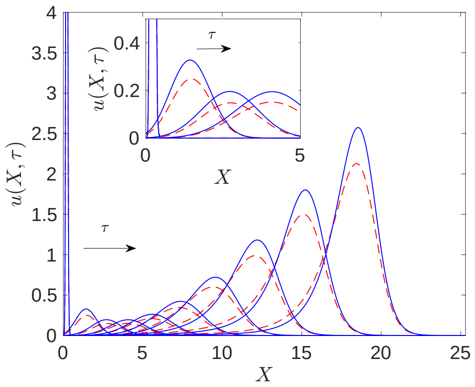


(a) Tip Cell Density

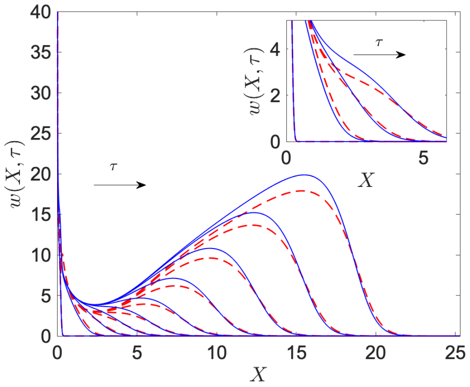


(b) Stalk Cell Density

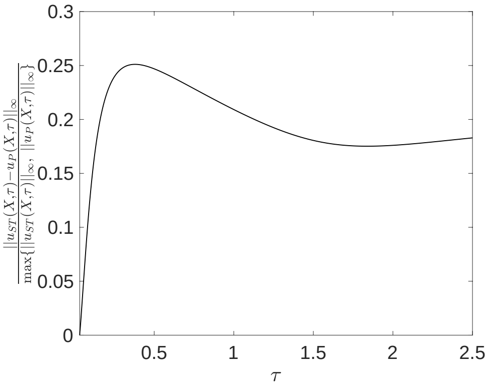


(c) $L_\infty$ norm, Tip Cell Relative Difference

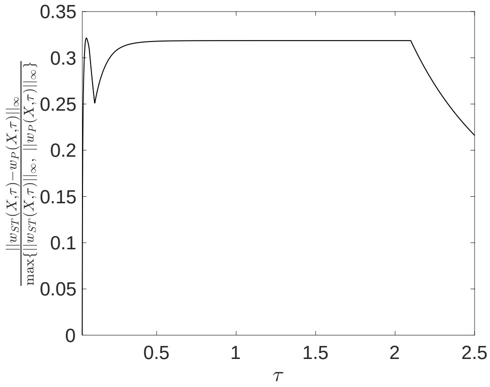


(d) $L_\infty$ norm, Stalk Cell Relative Difference

Figure 1: Numerical solutions of the ST–PDE and P–PDE systems showing how (a) the tip cell density, $u(X,\tau)$, and (b) the stalk cell density, $w(X,\tau)$, evolve over times $t = 0.2\lambda, 2.4\lambda, ..., 2.5$, with $\lambda = 0.16$. The value of $D$ was increased from $10^{-3}$ to $10^{-1}$, while all other values and initial conditions are the same as those used to generate Figure 3 of the main text (this corresponds to an increase in $\epsilon$ from $10^{-3/2}$ to about 0.32). The insets in the top two panels show a zoomed-in view of the results for the times $\tau = 0.2\lambda$, $2.4\lambda$, $4.6\lambda$, and $6.8\lambda$. The bottom row of figures shows the maximum relative difference between (c) tip cell densities of the ST–PDE, $u_{ST}(X,\tau)$, and P–PDE, $u_P(X,\tau)$, and (d) stalk cell densities of the ST–PDE, $w_{ST}(X,\tau)$, and P–PDE, $w_P(X,\tau)$. Key: ST–PDE (dashed red lines), P–PDE (solid blue lines). The PDEs were simulated on $\tau \in [0.2\lambda, 2.5]$, $X \in [0, 20\sqrt{\frac{\lambda}{D}}]$.

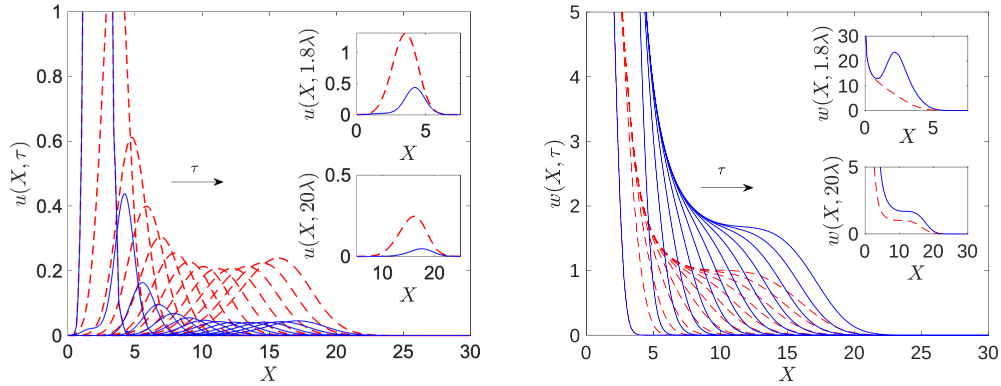


(a) Tip Cell Density (b) Stalk Cell Density

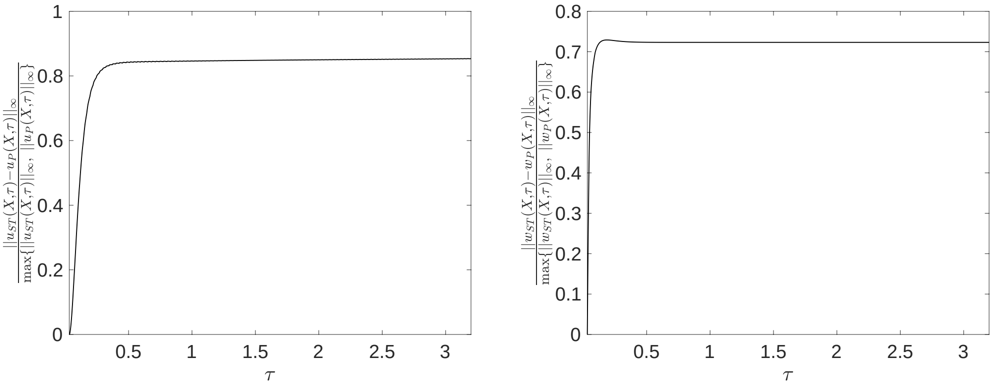


(c) $L_\infty$ norm, Tip Cell Relative Difference (d) $L_\infty$ norm, Stalk Cell Relative Difference

Figure 2: Numerical solutions of the ST–PDE and P–PDE systems showing how (a) the tip cell density, $u(X,\tau)$, and (b) the stalk cell density, $w(X,\tau)$, evolve over times $t = 0.2\lambda, 1.8\lambda, ..., 19.4\lambda$, with $\lambda = 0.16$. The parameter $\chi$ has been decreased from 0.4 to 0.04, while all other initial conditions and parameter values are the same as those used in Figure 3 of the main text (this corresponds to an increase in $\epsilon$ from $10^{-3/2}$ to about 0.32). The top insets in (a) and (b) show a zoomed-in view of the results at $\tau = 1.8\lambda$, while the bottom inset in those panels is a zoomed-in view of the results at $\tau = 20\lambda$. The bottom row of figures shows the maximum relative difference between (c) tip cell densities of the ST–PDE, $u_{ST}(X,\tau)$, and P–PDE, $u_P(X,\tau)$, and (d) stalk cell densities of the ST–PDE, $w_{ST}(X,\tau)$, and P–PDE, $w_P(X,\tau)$. Key: ST–PDE (dashed red lines), P–PDE (solid blue lines). The PDEs were simulated on $\tau \in [0.2\lambda, 20\lambda]$, $X \in [0, 20\sqrt{\frac{\lambda}{D}}]$.

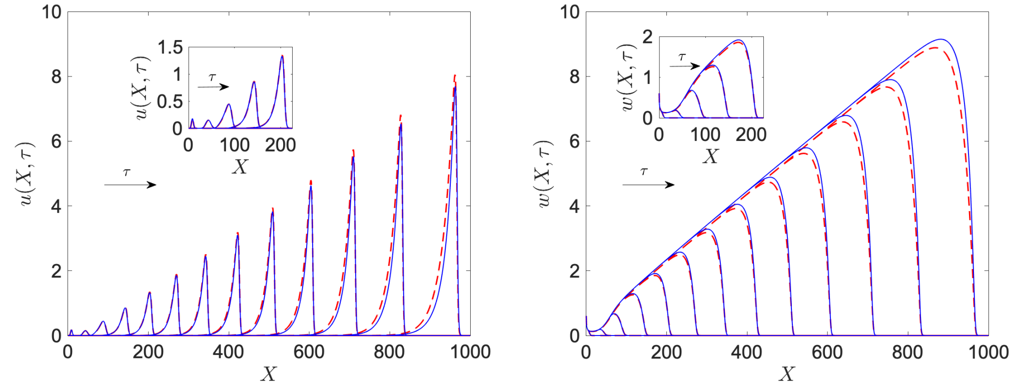


(a) Tip Cell Density (b) Stalk Cell Density

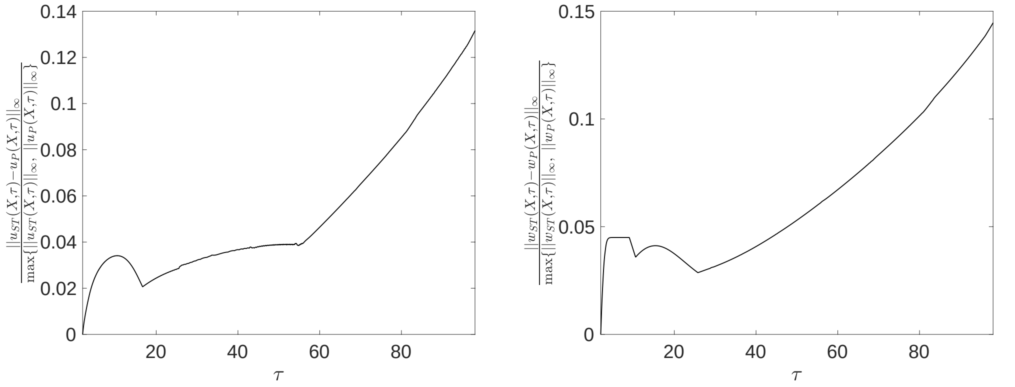


(c) $L_\infty$ norm, Tip Cell Relative Difference (d) $L_\infty$ norm, Stalk Cell Relative Difference

Figure 3: Numerical solutions of the ST–PDE and P–PDE systems showing how (a) the tip cell density, $u(X, \tau)$, and (b) the stalk cell density, $w(X, \tau)$, evolve over times $\tau = 0.2\lambda, 1.8\lambda, ..., 9.8\lambda$, when $\lambda$ is increased from from 0.16 to 10 (this corresponds increases in $\epsilon$ from $10^{-3/2}$ to about 0.32 and $\alpha$ from $10^{-3}$ to 0.0625). Other parameter values: as in Figure 3 of the main text. The insets in (a) and (b) show a zoomed-in view of the results at times $\tau = 0.2\lambda$, $1.8\lambda$, $2.4\lambda$, $4\lambda$, and $5.6\lambda$. The bottom row shows the maximum relative difference between (c) tip cell densities of the ST–PDE, $u_{ST}(X, \tau)$, and P–PDE, $u_P(X, \tau)$, and (d) stalk cell densities of the ST–PDE, $w_{ST}(X, \tau)$, and P–PDE, $w_P(X, \tau)$. Key: ST–PDE (dashed red lines), P–PDE (solid blue lines). The PDEs were simulated on $\tau \in [0.2\lambda, 9.8\lambda]$, $X \in [0, 1000]$.

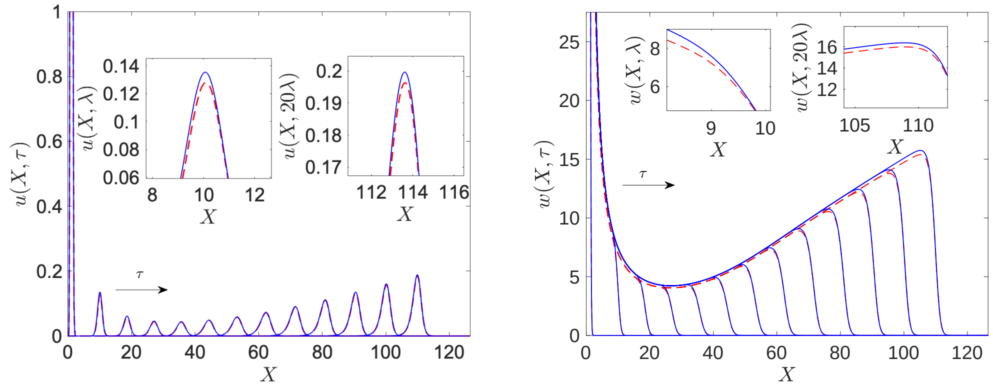


(a) Tip Cell Density (b) Stalk Cell Density

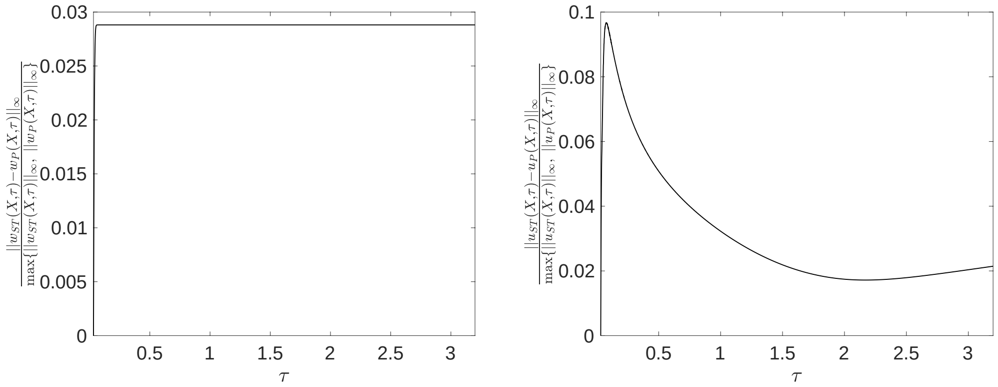


(c) $L_\infty$ norm, Tip Cell Relative Difference (d) $L_\infty$ norm, Stalk Cell Relative Difference

Figure 4: Numerical solutions of the ST–PDE and P–PDE systems showing how (a) the tip cell density, $u(X,\tau)$, and (b) the stalk cell density, $w(X,\tau)$, evolve over times $\tau = 0.2\lambda, 1.8\lambda, ..., 19.4\lambda$, with $\lambda = 0.16$. The parameter $a_e$ was increased from 0.0391 to 1, while keeping all other initial conditions and parameter values the same as those used to generate Figure 3 of the main text (this corresponds to an increase in $\beta$ from 39.1 to 1000, while $\epsilon = 10^{-3/2}$ and $\alpha = 10^{-3}$ remain constant). The left inset in (a) and (b) shows a zoomed-in view of the solution at $\tau = \lambda$, while the right inset in those panels shows a zoomed-in view of the solution at $\tau = 20\lambda$. The bottom row of figures shows the maximum relative difference between (c) tip cell densities of the ST–PDE, $u_{ST}(X,\tau)$, and P–PDE, $u_P(X,\tau)$, and (d) stalk cell densities of the ST–PDE, $w_{ST}(X,\tau)$, and P–PDE, $w_P(X,\tau)$. Key: ST–PDE (dashed red lines), P–PDE (solid blue lines). The PDEs were simulated on $\tau \in [0.2\lambda, 20\lambda]$, $X \in [0, 10\sqrt{\frac{\lambda}{D}}]$.

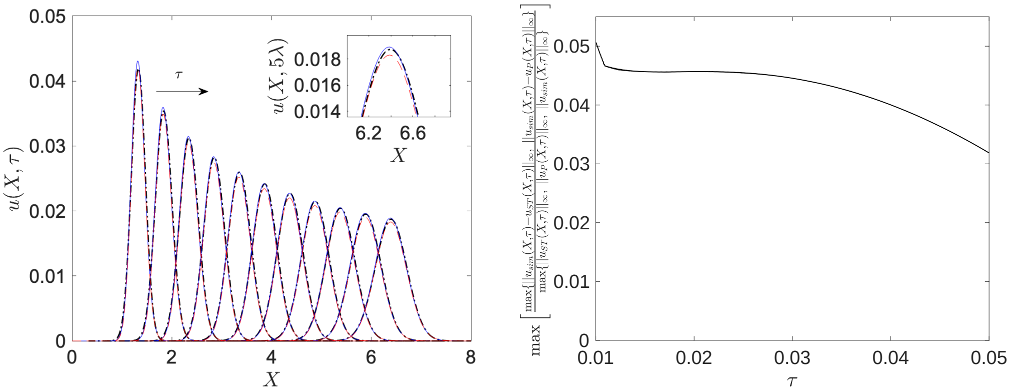


(a) Tip Cell Solution

(b) $L_\infty$ norm, Tip Cell Relative Difference

Figure 5: Self-similar solution given by equation (20) of the main text in (a) the independent variable $X$ for $\tau = 0.2\lambda$, $0.6\lambda$, ..., $5\lambda$, where $\lambda = 0.01$. In order to better compare the self-similar solution to the ST–PDE and P–PDE results, we set the maximum value of the self-similar solution at $\tau = 0.2\lambda$ equal to that of the ST–PDE and P–PDE tip cell densities at $\tau = 0.2\lambda$. The inset in (a) shows a zoomed-in view of the results at $\tau = 5\lambda$. In (b), we have plotted the relative error between the similarity solution and the P–PDE solution. Key for (a): P–PDE solutions (solid blue lines), ST–PDE solutions (dashed red lines), solution to equation (19) of the main text (dashed-dot black line).

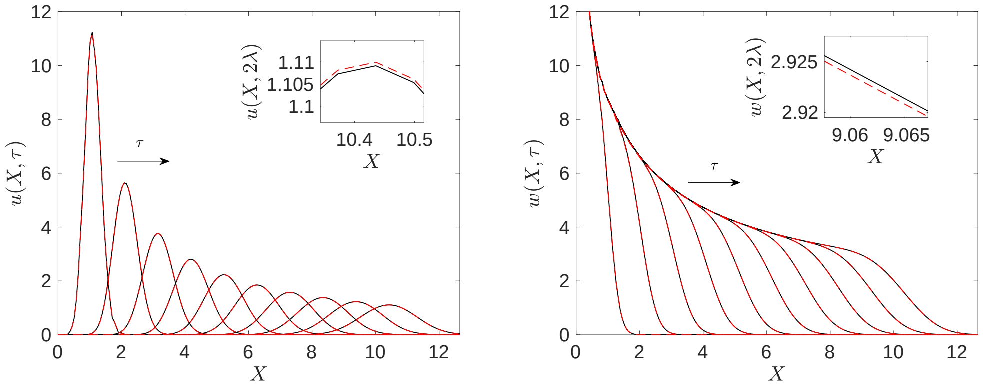


(a) Tip Cell Density

(b) Stalk Cell Density

Figure 6: Numerical solution of the leading order dynamics showing how (a) the tip cell density, $u(X,\tau)$, and (b) the stalk cell density, $w(X,\tau)$, evolve over times $\tau = 0.2\lambda, 0.4\lambda, \ldots, 2\lambda$, with $\lambda = 0.16$, along with results from the original P–PDE (the independent variables of the inner solution have been transformed back into the unscaled variables $X$ and $\tau$ for ease of comparison). The inset in both panels shows a zoomed-in view of the results at time $\tau = 2\lambda$. Key: P–PDE solution (solid black lines), leading order solutions (dashed red lines). Parameter values: $D = 10^{-3}$, $\chi = 0.4$, $a_n = 1$, $a_e = 0.0391$, $\mu = 160$ (this corresponds to $\epsilon = 10^{-3/2}$, $\alpha = 10^{-3}$, and $\beta = 39.1$). The PDEs were simulated on $\tau \in [0.2\lambda, 2\lambda]$, $X \in [0, \sqrt{\frac{\lambda}{D}}]$. P–ABM solutions at $\tau = 0.2\lambda$, column averaged in the $y$-direction, were used as initial conditions.
